# The application and sustainable development of coral in traditional medicine and its chemical composition, pharmacology, toxicology, and clinical research

**DOI:** 10.3389/fphar.2023.1230608

**Published:** 2024-01-03

**Authors:** Mengtian Han, Zhongyuan Wang, Yiye Li, Yinglian Song, Zhang Wang

**Affiliations:** ^1^ College of Pharmacy, Chengdu University of Traditional Chinese Medicine, Chengdu, China; ^2^ State Key Laboratory of Southwestern Chinese Medicine Resources, Chengdu University of Traditional Chinese Medicine, Chengdu, China; ^3^ College of Ethnomedicine, Chengdu University of Traditional Chinese Medicine, Chengdu, China

**Keywords:** coral, traditional medicine of China, chemical constituents, pharmacology, toxicology, clinical application

## Abstract

This review discusses the variety, chemical composition, pharmacological effects, toxicology, and clinical research of corals used in traditional medicine in the past two decades. At present, several types of medicinal coral resources are identified, which are used in 56 formulas such as traditional Chinese medicine, Tibetan medicine, Mongolian medicine, and Uyghur medicine. A total of 34 families and 99 genera of corals are involved in medical research, with the Alcyoniidae family and *Sarcophyton* genus being the main research objects. Based on the structural types of compounds and the families and genera of corals, this review summarizes the compounds primarily reported during the period, including terpenoids, steroids, nitrogen-containing compounds, and other terpenoids dominated by sesquiterpene and diterpenes. The biological activities of coral include cytotoxicity (antitumor and anticancer), anti-inflammatory, analgesic, antibacterial, antiviral, immunosuppressive, antioxidant, and neurological properties, and a detailed summary of the mechanisms underlying these activities or related targets is provided. Coral toxicity mostly occurs in the marine ornamental soft coral Zoanthidae family, with palytoxin as the main toxic compound. In addition, nonpeptide neurotoxins are extracted from aquatic corals. The compatibility of coral-related preparations did not show significant acute toxicity, but if used for a long time, it will still cause toxicity to the liver, kidneys, lungs, and other internal organs in a dose-dependent manner. In clinical applications, individual application of coral is often used as a substitute for orthopedic materials to treat diseases such as bone defects and bone hyperplasia. Second, coral is primarily available in the form of compound preparations, such as Ershiwuwei Shanhu pills and Shanhu Qishiwei pills, which are widely used in the treatment of neurological diseases such as migraine, primary headache, epilepsy, cerebral infarction, hypertension, and other cardiovascular and cerebrovascular diseases. It is undeniable that the effectiveness of coral research has exacerbated the endangered status of corals. Therefore, there should be no distinction between the advantages and disadvantages of listed endangered species, and it is imperative to completely prohibit their use and provide equal protection to help them recover to their normal numbers. This article can provide some reference for research on coral chemical composition, biological activity, chemical ecology, and the discovery of marine drug lead compounds. At the same time, it calls for people to protect endangered corals from the perspectives of prohibition, substitution, and synthesis.

## Graphical Abstract

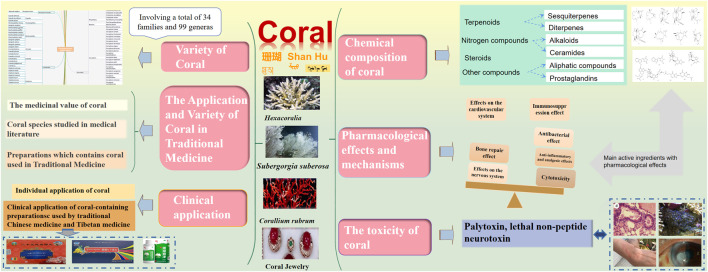

## 1 Introduction

Marine biological resources are abundant, and coral is a common organism in the ocean. It is a low-level invertebrate of the ocean, belonging to the phylum Coelenterata and the class Coralis. Coral mainly lives in tropical oceans and has a wide variety and distribution. There are over 6,100 species of coral worldwide and 719 species in China (Li T. T., 2010). Corals can be divided into Hexacorallia and Octocorallia (Xu, 2016). Corals are known as “sea flowers” and are a type of aquatic coelenterate. Their population is dendritic, branching and spreading like fans, with fine branches. Their surface contains many hydra bodies called anthozoan polyps. Their body is hemispherical in shape, with eight feathered tentacles on top. The tentacles have a mouth in the center, and the insect body can secrete limestone to form bones. White is better than snow; red is similar to blood; green is similar to jade, and yellow is similar to gold. Coral naturally grows in the sea, with strange shapes and unparalleled beauty (National Compilation of Chinese Herbal Medicine, 1996). The main compound of coral is calcium carbonate, which also contains a series of elements such as iron, manganese, copper, and strontium, as well as chitin and organic acids. Corals are commonly white, whereas gemstone-grade corals are red, pink, and orangey red, with a small amount of black and blue. The color of coral is due to its content of approximately 1% iron oxide and organic matter. With red as the top grade, red coral is as red as fire, known as the “fire tree” in ancient times. Its origin is in the deep sea of the Mediterranean and Atlantic oceans, and it is primarily used for jewelry, with the largest being used for carving figures, flowers, and birds, and other handicrafts (Wen, 2007). In India and Tibet of China, people use coral as a mascot for worship, often used to make Buddhist beads and decorate deities. In the West, coral is one of the three major organic gemstones, whereas in the East, coral symbolizes auspiciousness and happiness since ancient times. It also represents nobility and power and symbolizes happiness and eternity (Wen et al., 2007). The ancient Romans believed that coral played roles in disaster prevention, intelligence, hemostasis, and heat dissipation, which continued until this century (Hong, 2009).

Corals are distributed in the South China Sea, North China Sea, and East China Sea, among which the South China Sea is located in a tropical and subtropical zone and contains abundant coral biological resources. Since the 1980s, chemists have conducted in-depth research on corals in the South China Sea. At the beginning of the 20th century, the utilization of coral resources includes human bone substitutes, feed calcium filler, and a good source of calcium supply for the human body (Huang et al., 1997). With the rapid development of modern separation and identification methods and the increasing maturity of biotechnology, a large number of active substances have been isolated from marine organisms (Zhang G. et al., 2013), such as salicin with antibacterial activity and alkaloids with cytotoxic activity, which have been isolated from *Sinularia suberosa* on the side of the South China Sea (Qi et al., 2005). In addition, antitumor alkaloids have been obtained from *Ellisella curvata* on the side of the South China Sea (Zhang J. R., 2012). With the deepening of chemical research on natural products of soft coral and gorgonians^※^, thousands of compounds with dozens of structural skeletons have been discovered, including steroids, terpenoids, nitrogen-containing compounds, long-chain fatty acids, and long-chain alcohols. The diverse structures, unique molecular frameworks, and significant pharmacological activities of coral secondary metabolites fully demonstrate their potential medicinal value (Zhang W. et al., 2006; Shao et al., 2009a).

In the late 1960s, scholars and others discovered prostaglandin precursors with unique structures and strong physiological activity from gorgonian^※^, which further promoted coral chemistry research. The pharmacological activity of coral is also gradually being explored, which is primarily manifested in various aspects such as antitumor, anticancer, antioxidant, and anti-cardiovascular and in cerebrovascular system diseases. These pharmacological effects are mostly exerted by a single active substance extracted from coral bodies, while the bones of corals are mostly used as materials for bone transplantation and other applications (Wang et al., 2002b). Coral, as a medicinal material, has been recorded in detail in *The Compendium of Materia Medica* (1578 AD). It tastes sweet, and the property of the medicine is flat. It can improve eyesight, tranquilize the mind, and stop epilepsy. Coral is primarily used to treat corneal opacity. It also dissipates blood stasis. Coral powder can stop epistaxis. In the clinic, coral is also used in various compound preparations, such as Ershiwuwei Shanhu pills and Shanhu Qishiwei pills, which can restore nerve function and relieve pain. It is used in the treatment of albichoriasis, unconsciousness, body numbness, dizziness, brain pain, irregular blood pressure, headache, epilepsy, and various types of neuropathic pain. *The Compendium of Materia Medica* (1578 AD) records that corals are nontoxic, but according to literature reports, corals can release toxins, which are the second largest known deadly gas in the world, ultimately leading to toxic reactions such as muscle pain, four-limb weakness, and fainting. By contrast, in compound use, short-term use does not produce acute toxic reactions, but long-term use can cause damage to the liver, kidneys, and other organs.

In this review, we first conducted keyword searches on coral on academic websites such as PubMed, ScienceDirect, and CNKI and screened thousands of literature works related to medicine. Second, we conducted data mining to establish a database and finally extracted effective information for organization and analysis. This review discusses the use of coral in traditional medicine and its application in chemical composition, pharmacology, toxicology, and clinical research in the past two decades to provide important research data for the comprehensive development of marine biological resources, the discovery of drug lead compounds, the chemical ecological research of marine invertebrates, and the determination of organic synthetic chemical target compounds.

## 2 The application and variety of coral in traditional medicine

### 2.1 Coral species studied in the medical literature

**FIGURE 1:**
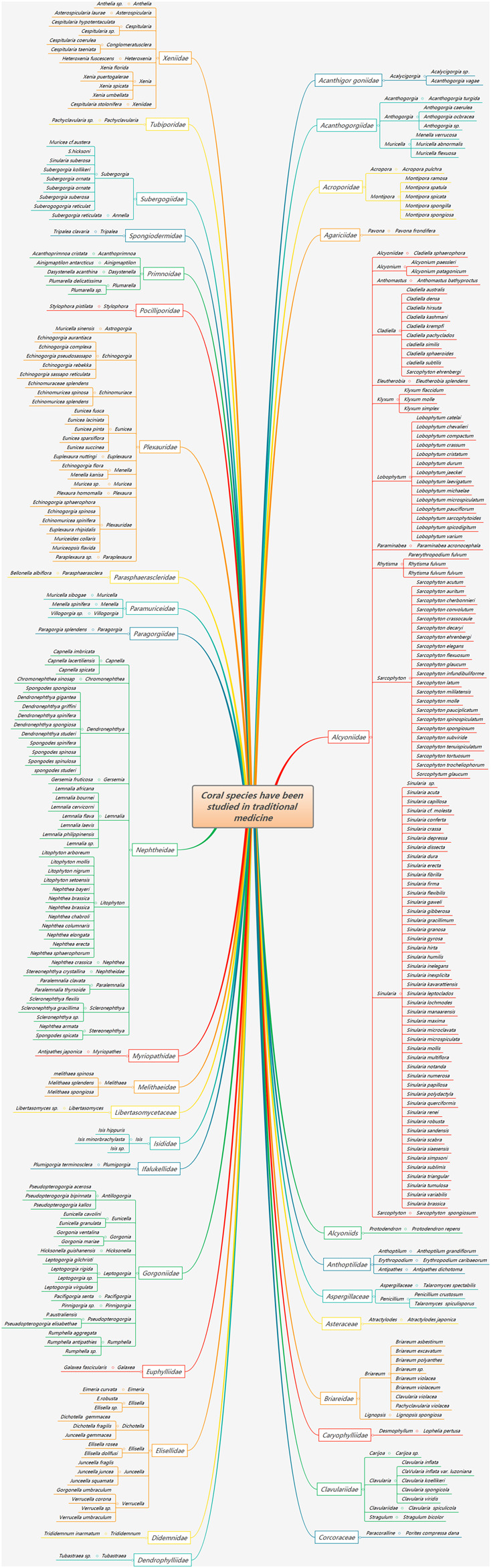
Coral species studied in the medical literature.

Tang Sujing mentioned in the *Newly Revised Materia Medica* in 659 AD that *Corallium rubrum* (Linnaeus), also known as red coral, Hong shan, Huo shu, and *Corallium japonicum* ^※^, belongs to the genus *Corallium* in the family Coralliidae. In addition, *Corallium japonicum* Kishinouye^※^ was included in the genus *Corallium* in the family Coralliidae in the *National Compilation of Chinese Herbal Medicine* (Second Edition). Zhuru, Ulan Shuru, and Shuru are recorded as Mongolian medicines. *Fossilia corrallium* is recorded as a Uyghur medicine in the *Dictionary of Chinese Ethnic Medicine*, which is mostly distributed in the Baihe Mahle River. It is commonly used to treat diarrhea, gastrointestinal bleeding, and neurasthenia. The *Dictionary of Traditional Chinese Medicine* also records *Corallium japonicum* Kishinouye^※^, which is recorded as *Corallium konojoi* ^※^ with the same name as that recorded in the *Chinese Traditional Chinese Medicine Resources*. *Corallium secundum* Dana^※^ and *Corallium elatius* Ridley are also recorded. The *Records of Chinese Traditional Chinese Medicine Resources* (*Part 2*) also records six species of coral, namely, *Porites nigrescens* Dana in the Poritidae family, *Porites* genus; *Antipathes* sp.; and the national first-class protected wild animals *Corallium japonicum* Kishinouye^※^, *Corallium elatius* Ridley, and *Corallium konojoi* Kishinouye^※^. In the past two decades, most of the coral species that have been studied in medicine belong to Alcyoniidae, Gorgonacea^※^, and Scleractinia^※^. After sorting, red coral is mostly used in medical records. Modern research on coral species is diverse, involving a total of 34 families and 99 genera. Corals in the Alcyoniidae, Nephtheidae, Plexauridae, Gorgoniidae^※^, Xeniidae, Elisellidae, Briareidae, Subergorgiidae, and Clavulariidae families are more common. *Sarcophyton* and *Sinularia* are research hotspots in Alcyconiidae, followed by *Dendronephthya*, *Litophyton*, and *Lemnalia* corals in the Nephtheidae family and by *Echinogorgia*, *Plexauridae*, and *Eunicea* corals in the Plexauridae family (Figure 1).

### 2.2 The medicinal value of coral

The records of coral can be traced back to the Three Kingdoms period (226–231 AD). Kangtai and Zhu Ying of the Eastern Wu Dynasty mentioned in their *Biography of Fu Nan* that “In the rising sea, the coral reef falls, and there is a rock at the bottom of the reef, and the coral grows on it” (Cao, 2012). Coral is used as a medicinal material, which was first recorded in the *Newly Revised Materia Medica* (659 AD) as “sweet, flat, and nontoxic” and primarily used for blood retention and corneal opacity. In addition, coral is ground to a powder and used to stop epistaxis. It grew in the South China Sea, resembling jade red, with many pores in the middle and some without pores. It can also be found in Persia and Sri Lanka. “*The General Introduction to the Essential Prescriptions of Zengguang and Zhiju*” (1208 AD) records that coral is effective in the removal of corneal opacity and cessation of bleeding in epistaxis. *Yue Hau zi* (908–923 AD) describes that coral can tranquilize the mind and stop epilepsy. *Oversea Materia Medica* (907–960 AD) records that coral is the main cause of blood stasis and wind epilepsy. The classic work *Compendium of Materia Medica* (1578 AD) points out that coral can treat corneal opacity. *Materia Medica Yanyi* (1116 AD) records that coral can be used to remove corneal opacity. *Compendium of Selected Essentials of Materia Medica* (1,644–1911 AD) describes that coral is primarily used for corneal opacity, blood stasis, and epistaxis. It can also improve eyesight, tranquilize mind, stop epilepsy, and drop and remove flying silk. In the *Second Edition of the National Compilation of Chinese Herbal Medicine* (*Volume 2*; compiled by the Compilation Team of the National Compilation of Chinese Herbal Medicine, 1996), coral is mentioned as red coral, with sweet and flat properties; it can tranquilize the mind, stop epilepsy, and improve eyesight, and it is primarily used in treating convulsions, stopping epilepsy, and removing corneal opacity. Traditional Chinese medicine books such as *Taiping Holy Prescriptions for Universal Relief* (992 AD), *Fangmai Zhengzong* (1749 AD), *Peng Family Miao Prescription*, and *Aquatic Product Nutrition and Medicinal Manual* all contain prescriptions made from red coral, which can remove corneal opacity in children, dizziness, epilepsy or palpitations, heart and lung congestion, persistent vomiting and bleeding, and water and fire burns (Lai et al., 2016).

Tibetans, Mongols, and Uyghurs also often use coral as a medicinal material for compatibility treatment. Coral Tibetan medicine, namely, Qiwuru, also known as Pazhuma, can clear liver heat and detoxify various toxins. It is primarily used to treat encephalopathy, liver disease, various fevers, and poisoning. The Mongolian medicine, namely, Shuru, which is also known as Zhuru and Ulan Shuru, can clear heat, detoxify toxins, and tranquilize the mind. It is primarily used to treat liver heat, lung heat, detoxify, toxic heat, stroke, and brain disease. The Uyghur medicine, namely, Bihe Marjiang, which is also known as Busai, can restore function and astringing sores, clear heat and inflammation, traete loose teeth, refresh the heart, please the mind, and stop bleeding and diarrhea. It is primarily used to treat damp heat or blood-related diseases. Li et al. (2015) found through experimental research that Mongolian Jiegu Medicine Water Pills have good therapeutic effects on fractures. A’naer Vigills can clear heat, restore function, and relieve itching. It has been used for various symptoms, such as itching, redness, swelling, and excessive vaginal discharge, caused by bacterial and fungal vaginitis in women. It is a commonly used Uyghur medicine preparation in clinical practice (Chen, 2011). Ershiwuwei Shanhu pills can intervene in the treatment of neurological diseases such as Alzheimer’s disease, cerebral infarction, and migraine (Zhou et al., 2019; Zhu et al., 2020; Jiaojia et al., 2022).

The use of coral in modern medicine is no longer limited to red coral. Jiang (2013) conducted an extraction experiment on the active ingredients of *Dichotella gemmacea* ^※^ and found that some of its diterpenoid compounds showed cytotoxicity to human lung pancreatic cancer cells (A549) and human osteosarcoma cells (MG63), and some of the compounds had antibacterial activity. Wu (2013) conducted a study on *Echinogorgia flora* and found that its sesquiterpene active ingredients showed a weak antiviral activity against influenza virus. Mahmoud et al. (2022) showed that the steroids and sesquiterpene of Red Sea soft corals showed evident activity on A549, MCF-7, and HepG2 cell lines. The chemical compounds in Scleractinia^※^ (Zhao et al., 2016) exhibit good biological activities, such as cytotoxic, antibacterial, insecticidal, and toxic effects on fish. At present, the corals used as medicinal materials include soft corals, gorgonians^※^, Scleractinia^※^, and red corals (Ai et al., 2006). Scleractinia^※^ have received little attention from chemists because they are primarily composed of calcareous bones, and the scarcity of red coral resources also limits their utilization. Therefore, active soft corals and gorgonians^※^ have become the first option for coral reef benthic research, and they are increasingly becoming popular biological species in modern marine natural product research (Xue, 2014).

### 2.3 Preparations that contain coral used in traditional medicine

Coral is used as a medicinal material, which has a long history in China. Ancient Chinese ancestors recognized the medicinal value of coral. Coral is primarily used in traditional Chinese medicine, Tibetan medicine, and Mongolian medicine, but the specific variety of coral is not clearly specified in the prescription. Red coral is primarily used as medicine, and the method of medicine includes the following steps: take the original medicinal material, remove impurities, wash and grind it into a fine powder, sieve to obtain an extremely fine powder, and dry it. The compatibility of its medication is shown in Table 1. It is primarily used to treat nervous system disease, chronic ulcers, and various heat syndromes. Traditional Chinese herbs and formulas often play a role in clearing heat, treating eye diseases, relieving chest and hypochondriac swelling and pain caused by diseases, and dissipating heat in the liver and gallbladder. Tibetan medicine is used to treat headache, epilepsy, and various types of neuropathic pain caused by albichoriasis. Apart from traditional Chinese medicine and Tibetan medicine, Mongolian medicine has a wide range of treatments, including various new and old fractures, soft tissue injuries, femoral head necrosis, and various edemas. Records in Uyghur medicine provide evidence for the treatment of various bacterial and fungal infections and trichomonal vulvovaginitis, causing itching, redness, and swelling of the genital area, as well as excessive vaginal discharge, in women.

**TABLE 1:** Preparations that contain coral used in traditional medicine (Lai et al., 2016).

Name of the preparation	Systems of traditional medicine	Indication	Source
Teling eye ointment	Traditional Chinese medicine	Swelling and pain of eyes, epidemic hemorrhagic conjunctivitis, marginal blepharitis, trachoma, and corneal opacity	Ministry of Health of the People’s Republic of China Drug standards Volume 14 of traditional Chinese medicine preparations
Jinniu eye ointment	Traditional Chinese medicine	Epidemic hemorrhagic conjunctivitis, marginal blepharitis, trachoma, eyes tear up in the wind, and external eye diseases such as Suyi	Ministry of Health of the People’s Republic of China Drug standards Volume 20 of traditional Chinese medicine preparations
Jinniu eye ointment	Traditional Chinese medicine	Epidemic hemorrhagic conjunctivitis, marginal blepharitis, trachoma, tears in wind and external eye diseases such as Suyi	New National traditional Chinese patent medicines and simple preparations 2nd Edition
Babao Boyun powder	Traditional Chinese medicine	Swelling and pain of eyes and pterygium	National Prescription Collection of Traditional Chinese Medicine (Nanjing Formula)
Babao Guangming powder	Traditional Chinese medicine	Swelling and pain of eyes, inflammation of the conjunctiva, photophobia and tears, and wind–heat congestion	National Prescription Collection of Traditional Chinese Medicine (Sha shi Formula)
Babao Ruiren plaster	Traditional Chinese medicine	Corneal opacity and xerophthalmia	System of Ophthalmology Volume 6
Babao eye ointment	Traditional Chinese medicine	Epidemic hemorrhagic conjunctivitis, swelling and pain of eyes, corneal opacity, pterygium, photophobia, and marginal blepharitis	National Prescription Collection of Traditional Chinese Medicine (Tianjin Formula)
Babao eye ointment	Traditional Chinese medicine	Epidemic hemorrhagic conjunctivitis, swelling, pain, stickiness, corneal opacity, photophobia, and tears	Traditional Chinese Medicine Formula Preparation
Bo feng yun plaster	Traditional Chinese medicine	Corneal opacity, epidemic hemorrhagic conjunctivitis, pterygium, and bloodshot eyes	Yixue Rumen Roll 7
Boyi Zijinplastter	Traditional Chinese medicine	All kinds of acute conjunctivitis and blood–membrane barriers	Outline for Men’s Diseases Roll 101
Dajin pill	Traditional Chinese medicine	Sputum fire-burnt diaphragm, wind damp phlegm, asthenic disease, and timidity syndrome	Zunsheng Bajian Volume 18
Dianyan Qibao powder	Traditional Chinese medicine	Wind heat rush up and acute conjunctivitis	General Medical Collection of Royal Benevolence Roll 105
Fo Bao Dan (Saizhen powder)	Traditional Chinese medicine	Throat poisoning and throat ulcers	Guide Book for Laryngology Roll 1
Gengong Chuhai pills	Traditional Chinese medicine	All symptoms of diphtheria	Complete Collection of Diphtheria
Hongding eye ointment	Traditional Chinese medicine	Hyperemia of bulbar conjunctiva, marginal blepharitis, and epidemic hemorrhagic conjunctivitis	Prescriptions for Universal Relief Roll 77
Wiping teeth white quartz powder (white quartz powder)	Traditional Chinese medicine	Tooth whitening	General Records of Holy Universal Relief Roll 121
Keming Liangyan ointment	Traditional Chinese medicine	Swelling and pain of eyes, bloodshot eye, and obstruction	National Prescription Collection of Traditional Chinese Medicine (JiNan Formula)
Luma Baoyuan pill	Traditional Chinese medicine	Supporting Yang and suppressing Yin and supplementing benefits and prolonging Years	Prescriptions for Universal Relief Roll 223
Qibao powder	Traditional Chinese medicine	Corneal opacity	A Profound Treatise on Eye Diseases
Zhenzhu powder	Traditional Chinese medicine	Corneal opacity	Zhenzhu Shibaosan (Surgical Prescription and Extraordinary Prescription roll 2)
Qishiwei Songshi pills	Tibetan medicine	Chest and hypochondriac pain, vomiting, hiccup, and loss of appetite caused by liver stagnation and stagnation and heat stasis	National Standard Compilation of Proprietary Chinese Medicines Internal Medicine Hepatobiliary Volume
Sanshiyiwei Songshi pills	Tibetan medicine	Acute and chronic hepatitis caused by diseases and heat in the liver and gallbladder	National Standard Compilation of Proprietary Chinese Medicines Internal Medicine Hepatobiliary Volume
Sishierwei Shugan capsules	Tibetan medicine	Damp heat in the liver and gallbladder, hypochondriac pain caused by stagnation and blood stasis, and abdominal distension; acute and chronic hepatitis B with the above symptoms	National Standard Compilation of Proprietary Chinese Medicines Internal Medicine Hepatobiliary Volume
SareShisanweiPengniao Pills	Tibetan medicine	Apoplexy, oral and eye deviation, numbness and paralysis, vasculitis, tenosynovitis, limb joint dysfunction, and leprosy caused by albichoriasis	Tibetan medicine in the Drug Standards of the Ministry of Health Volume Ⅰ
Ershiwuwei Songshi Pills	Tibetan medicine	Liver depression and stagnation, blood stasis, liver poisoning, liver pain, liver cirrhosis, liver effusion, and various acute and chronic hepatitis and cholecystitis	Pharmacopoeia of the People’s Republic of China 2020 Volume Ⅰ
Ershiwuwei Shanhu pills	Tibetan medicine	Albichoriasis, unconsciousness, body numbness, dizziness, brain pain, irregular blood pressure, headache, epilepsy, and various neuropathic pain conditions	Pharmacopoeia of the People’s Republic of China 2020 Volume Ⅰ
Ershiwuwei Shanhu capsules	Tibetan medicine	Albichoriasis, unconsciousness, body numbness, dizziness, brain pain, irregular blood pressure, headache, epilepsy, and various neuropathic pain conditions	New Drug Regularization Criteria Volume 83
Hupo powder	Tibetan medicine	Weary eyes and corneal opacity	Precious Book of Ophthalmology Roll 3
Ruyi Zhenzhu powder	Tibetan medicine	Plague, heat enters the choroid and cannot be cured for a long time, rheumatoid arthritis, scrofula, contractures, renal vein damage, and albichoriasis	Lantab
Ershisanwei chen powder	Tibetan medicine	Cough with gray phlegm, red phlegm, yellow phlegm, and other symptoms	Shanhu Zan
Shibawei Jiangjun powder	Tibetan medicine	Albichoriasis	Shanhu Zan
Jing ying pills	Tibetan medicine	Albichoriasis, xila wusu, cerebral hemorrhage, muscle and tendon pain, and other symptoms	Linzheng Zhaji
Sishiwei Jiangjun powder	Tibetan medicine	Various poisoning symptoms	Ganlu Baijing
Mingmu pills	Tibetan medicine	Various febrile liver diseases and various eye diseases	Linzheng Zhaji
SareShisanweiPengniao pills	Tibetan medicine	Ocular deviation, numbness and paralysis caused by albichoriasis as well as vasculitis, tenosynovitis, and disadvantageous limb joints	Tibetan Medicine Standards
Sishibawei Jiedu powder	Tibetan medicine	Poisoning attacks such as self-poisoning, solid poisoning, visible poisoning, contact poisoning, sunlight poisoning, and oral poisoning	Summary of Ganlu Prescription
Shibawei Xijiao powder	Tibetan medicine	Albichoriasis	Summary of Ganlu Prescription
Coral Bone Joining pill (Sunrise Tu Uril)	Mongolian medicine	Various new and old fractures, soft tissue injuries, and femoral head necrosis	Essence of Hundred Therapeutic Prescriptions
Jiuwei Hailuo powder	Mongolian medicine	Panic, palpitations, fever, heart adhesion, dry mouth and tongue, and other symptoms	Yiyao yuedi
Shisanwei Ying pill	Mongolian medicine	Albichoriasis, cerebral hemorrhage, hemiplegia, and poisoning	Selected Compilation of Mongolian Medicine
Zhachong Shisanwei pill	Mongolian medicine	Hemiplegia, left paralysis and right paralysis, distorted mouth and eyes, numbness in limbs, unfavorable waist and legs, unclear speech, muscle and bone pain, nerve paralysis, rheumatism, and joint pain	Mongolian Medicine in the Drug Standards of the Ministry of Health Volume
Ershiwei Huangjin powder	Mongolian medicine	Albichoriasis	Mongolian Medicine Golden Chamber
Bianbao pills	Mongolian medicine	Various edema syndromes	Mongolian Medicine Golden Chamber
Lianchuang powder	Mongolian medicine	All kinds of long-term sores do not heal	Guanzhe Zhixi
Shiwei Baohui powder	Mongolian medicine	Various edema syndromes	Mongolian Medicine Golden Chamber
Shiqiwei Jinhui pills	Mongolian medicine	Scrofula and black Hiraousu disease	Selected Prescriptions for Mongolian Medicine
Shibaweiguan pills	Mongolian medicine	Wind cold, muscle pain, numbness in limbs, choroidal diseases	Selected Prescriptions for Mongolian Medicine
Jiuwei Xionghuang powder	Mongolian medicine	Seasonal heat, plague and toxin, acute fire and convulsive wind, and various fever symptoms	Mongolian Medicine Prescription
Sishiwei Chenxiang powder	Mongolian medicine	Spermatorrhea	Mengyi Miaofang
Ershiwuwei Songshi pills	Mongolian medicine/Tibetan medicine	Various liver diseases	Clinical Experience in Mongolian Medicine
Shiwuwei Zhenzhu powder	Mongolian medicine/Tibetan medicine	In an abject state of mental confusion and forgetfulness	Clinical Experience in Mongolian Medicine
Shiwuweirupeng pills	Mongolian medicine/Tibetan medicine	Rheumatoid disease	Mongolian Medicine Golden Chamber
Shanhu qishiwei pill	Mongolian medicine/Tibetan medicine	Cerebral thrombosis, cerebral hemorrhage, coronary heart disease, limb paralysis, tachycardia or bradycardia, hypertension, poliomyelitis, epilepsy, and various types of neuritis. Particularly, effective for brain, nervous, and heart diseases	Mongolian Medicine in the Drug Standards of the Ministry of Health Volume
A’naer Vigills	Uyghur medicine	Various bacterial, fungal, trichomonal vulvitis, and vaginitis can cause itching, redness, and swelling of the genital area in women, as well as excessive vaginal discharge	Uyghur Medicine in the Drug Standards of the Ministry of Health Volume
GangKangMuKuLi tablets	Uyghur medicine	Hemorrhoids, cluneal cleft, and hematochezia	Uyghur Medicine in the Drug Standards of the Ministry of Health Volume
Poison symptom drug powder	—	Various poison formulations	Jin Yaoshi

## 3 Chemical composition of coral

In recent years, Chinese scholars have made important contributions to the research of international marine natural products. In 1980, Su Jingyu first isolated two new types of diterpenoid dimers with double fourteen-membered cyclic carbon frameworks from soft corals (Xue, 2014). Weinheimer and Washecheck (1969) first discovered abundant and highly active prostaglandin-like compounds from gorgonians^※^. These research results have aroused great interest in the study of coral chemical composition. After decades of research exploration and development, a large number of structurally novel and biologically active compounds have been discovered and determined from corals. Each type of compound contains many compounds with different structures, such as terpenoids, alkaloids, steroids, macrolides, quinones, polyethers, flavonoids, and peptides (Li R., 2012). The following sections provide an explanation of the chemical composition of corals based on different structural types.

### 3.1 Terpenoids

Terpenoids are the most abundant and diverse class of compounds in coral, and terpenoids with a new skeleton are constantly being discovered. Its pharmacological screening shows strong biological activity (Zhang and Guo, 2003; Liu, 2017). Therefore, the isolation and identification of terpenoids have always been the focus and hotspot of coral chemistry research. After sorting out and analyzing the literature, the primary terpenoid compounds are sesquiterpene and diterpenes, in addition to semiterpenoids and triterpenes.

#### 3.1.1 Sesquiterpenes

**FIGURE 2:**
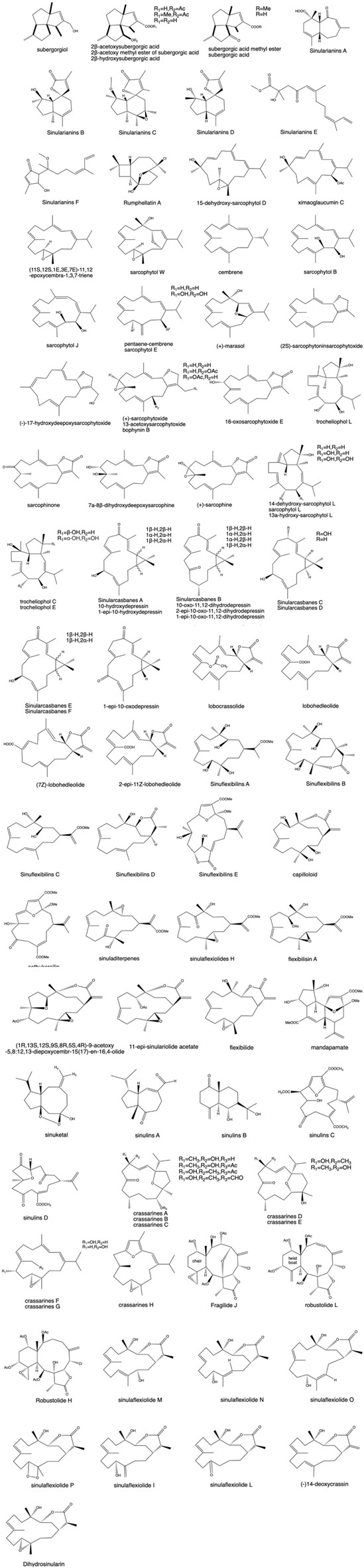
(Continued). Some pharmacologically active terpenoids extracted from coral.

Sesquiterpenes are an important class of terpenoids that are widely distributed in terrestrial fungi, higher plants, insects, and marine organisms such as soft corals. In addition to the earlier discovery of guaiacane and furan sesquiterpenes, sesquiterpene also contains africanne, capnellane, and illudalane (He, 2013). Wang et al. (2002a) isolated subergorgiol and 2β-acetyl subergorgic acid with a unique angular triquetane structure from the Taiwanese soft coral *S. suberosa*, in which subergorgiol exhibited moderate cytotoxicity against HeLa tumor cells. Menecubebane B, the known compound analog isolated from gorgonian^※^ *Menella* sp., showed moderate cytotoxicity against Eca9706 and HeLa cell lines with semi-inhibitory concentration values of 20.8 and 30.6 μM, respectively. In the coming year, Ngoc et al. (2017a) extracted and identified four sesquiterpenes, namely, nanolobatols A and B and sinularianins B and D, in the Vietnamese soft coral *Sinularia nanolobata*. Sinularianins B and D were similarly extracted from *Sinularia* sp. (Chao et al., 2006; Yang et al., 2013). A novel chlorine-containing carbon-deficient sesquiterpene was isolated from Taiwan gorgonian^※^, and this compound showed inhibitory effects on Gram-negative bacteria (Figure 2) (Sung et al., 2007).

#### 3.1.2 Diterpenes

Many diterpenes show strong biological activities, so diterpenoids have remained a focus and hotspot for research in the past few years. Diterpenes are the most abundant and diverse structural types in corals, and the most common and diverse diterpene is cembrane, which is characterized by an isopropyl and three methyl substitutions in the tetradecane ring. Other diterpenes include eunicellin, casbane, biflorane, briarellin, dolabellane, lobane, sarcodictyins, and xenia (Shao et al., 2009b). Li J. F. et al. (2022) extracted 20 sissonane-type diterpenes from *Sarcophyton glaucum*. The *Sinularia* genus is rich in diterpenes. As isolated from the extract of CH_2_Cl_2_/EtOH in it, 18 sesquiterpenes such as sinoflexibilins A–F were identified (Yin et al., 2013; Jiang et al., 2019a), and two sinulins C and D (Qin et al., 2018) were isolated from the CH_2_Cl_2_/C_2_H_5_OH extract of *Sinularia* sp. Some of the compounds exhibit some degree of cytotoxicity against A549 and HL-60 cells or exert anti-inflammatory effects through inducible nitric oxide synthase (iNOS) and cyclooxygenase-2 (COX-2) expression (Chao et al., 2011a). Wang et al. (2010) first discovered a new chlorinated briarane (fragilide J) and two chlorinated briaranes (robustolide L and robustolide H) from *Junceella fragilis* ^※^ and *Ellisella robusta* ^※^ *.* Jiao-Jiao Xu isolated 10 sissonane diterpenes from soft coral *Sinularia flexibilis* samples, and the inhibitory effects of each monomer compound on LPS-induced NO release from RAW 264.7 cells were examined using the Griess method at noncytotoxic doses. The results showed that the compounds had some inhibitory effects on NO production (Xu, 2016).

### 3.2 Steroids

**FIGURE 3:**
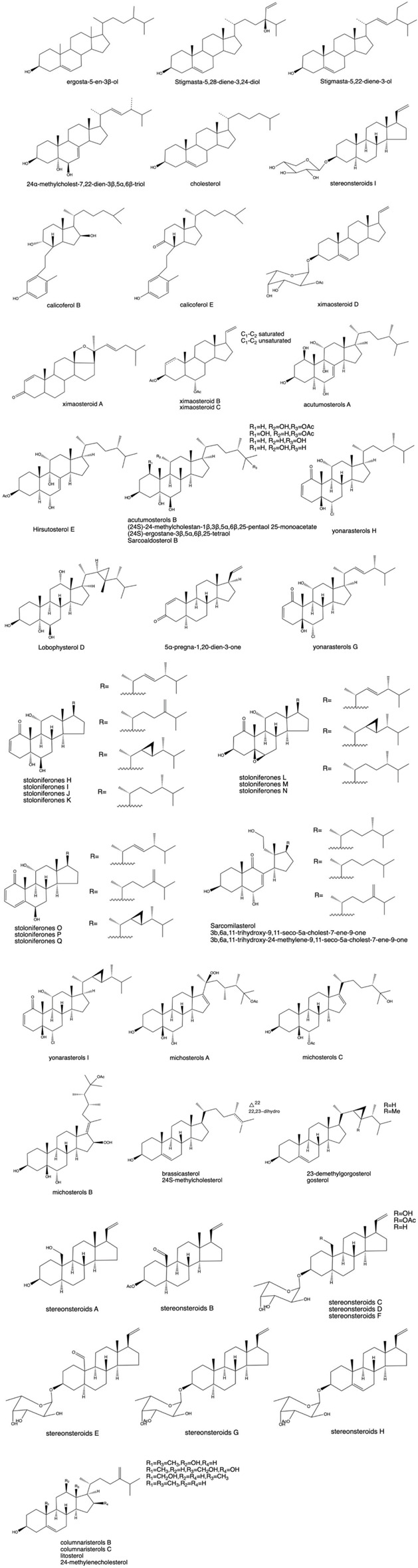
(Continued). Some pharmacologically active steroids extracted from coral.

As shown in Figure 3, steroids are a class of biologically active compounds in corals, particularly pregnane, cholestane, and ergosterone. It has received considerable attention because of its structural diversity and remarkable biological activity (Liu, 2017). Sterols are abundant in corals, and the structure is more complex because of the diversification of the sterol side chain structure and the different degrees of oxidation (Ai et al., 2006). Seven new cleaved ring sterols with C_9,11_ breaks and C_22_ hydroxylation were isolated for the first time from *Tripalea clavaria* collected from the South Atlantic Ocean in 2006, as determined by wave spectroscopy and the Mosher method, and further studies revealed that some of their substances showed some inhibitory activity against *Staphylococcus aureus*. Four bioactive sterols with anti-inflammatory, antibacterial, antioxidant, antitumor, and antitubercular properties were isolated from *J. fragilis* ^※^ from Sanya, Hainan (Wen et al., 2007). Subsequently, two sterols were isolated from the CH_2_Cl_2_/C_2_H_5_OH extract of this coral (Qi et al., 2004). For the first time, two B-ring open-loop sterols were isolated from the Chinese small pointed gorgonian *Muricella sinensis* (Verril1) ^※^ from the South China Sea. During bioactivity screening, calicoferol E was found to show inhibitory activity against protein tyrosine phospholipase 1B (PTP1B), with an IC_50_ value of 27.28 μM (Yan et al., 2005).

### 3.3 Nitrogen compounds

**FIGURE 4:**
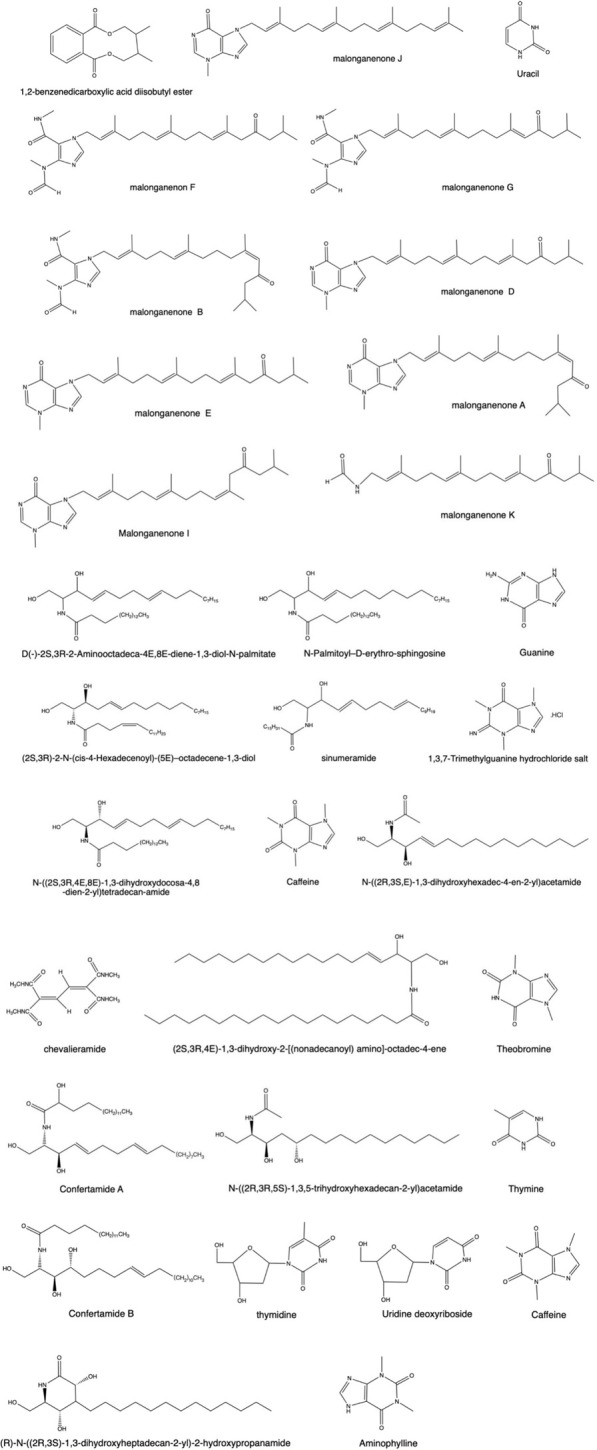
Some pharmacologically active nitrogen compounds extracted from coral.

The nitrogenous compounds in corals primarily include ceramides and alkaloids (deoxythymidine, thymine, methyluracil, and urea). They generally exhibit antifungal, antibacterial, and cytotoxic activities. Such compounds can also inhibit acetylcholestan-converting protease, thereby providing an alternative lead compound for the development of therapeutic drugs for atherosclerosis and other cardiovascular diseases (Ai et al., 2006). Zhang J. R. (2012) isolated 16 alkaloids (nine diterpene alkaloids, including three new diterpene alkaloid compounds) and five ceramides from *E. robusta* and *E. curvata* of gorgonian^※^. A preliminary evaluation of the antitumor activity at the cellular level was carried out, from which four diterpene alkaloids were screened to show strong cytotoxicity against HeLa and K562 cancer cells, and the enzymatic activity inhibition was evaluated by enzyme-linked immunosorbent assay (ELISA). The activity results showed that diterpene alkaloid malonganenone D had a strong inhibitory effect on the enzymatic activity of c-Met. The ceramide N-1-hydroxymethyl-2-hydroxy-(E, E)-3,7-heptadecadienylhexadecanoamide (Liu et al., 2001), thymine, and uracil were isolated from *Acropora pulchra* ^※^ (Brook; Xu et al. (2003)). In addition to different corals, such as *Litophyton arboreum* (Abou El-Kassem et al., 2018) and *Junceella juncea* ^※^ (*Pallas*; Krishna et al. (2004)), *Lobophytum chevalieri* (Li et al., 1989) has bioactive ceramides. The structure diagram is shown in Figure 4.

### 3.4 Other compounds

**FIGURE 5:**
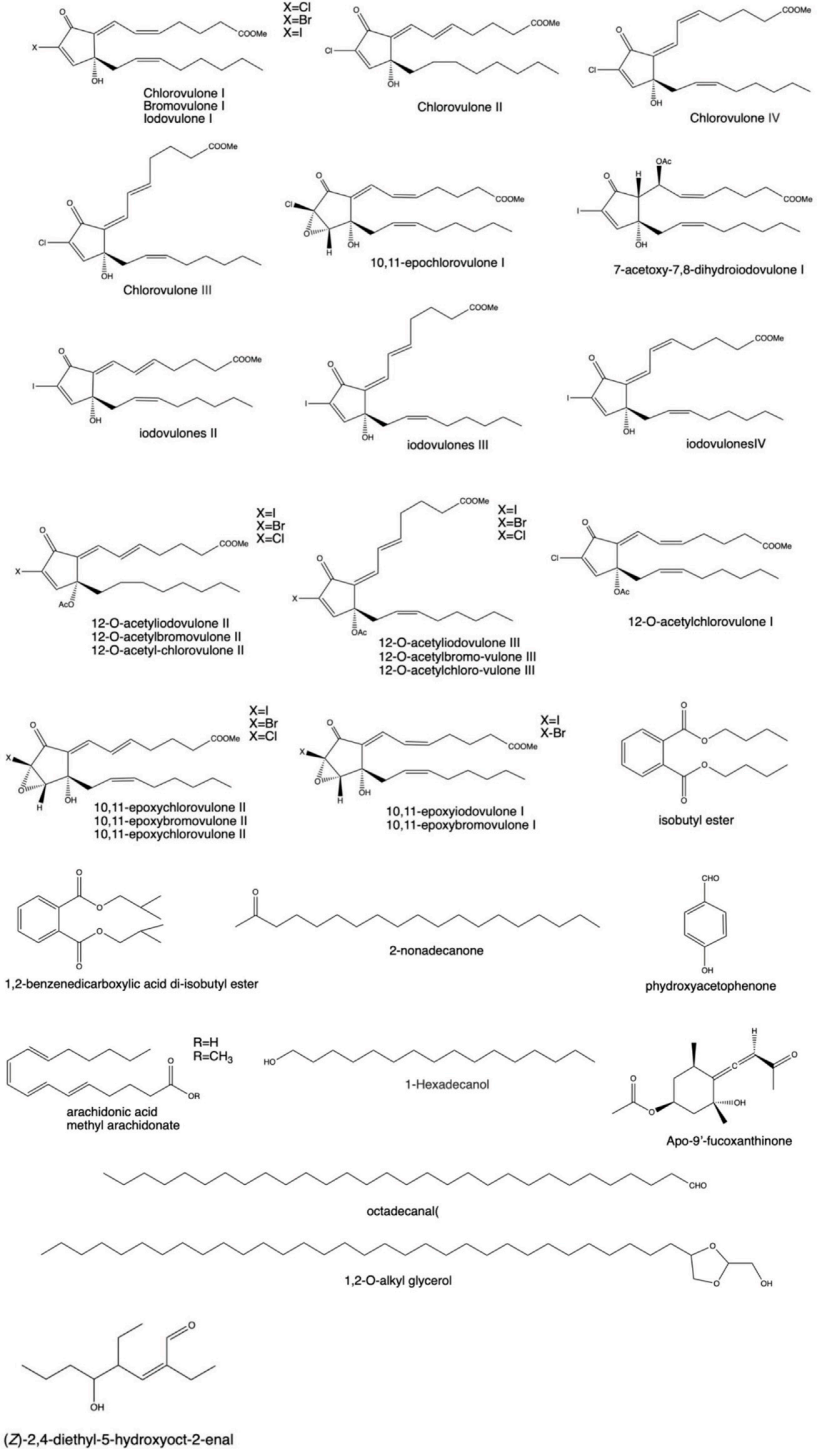
Some pharmacologically active compounds extracted from coral.

As shown in Figure 5, aliphatic compounds (long-chain fatty acids, long-chain aliphatic alcohols, and the aldehydes and esters they form) and prostaglandins were also extracted from different corals (Watanabe et al., 2003; Reina et al., 2013; Hurtado et al., 2020). According to the literature, a large amount of batyl alcohol has a pharmacological effect of raising leukocytes, which is extracted from coral and has been widely used in clinical practice (Ma, 2008; Zhao et al., 2011; Sun, 2012; Xue et al., 2014). Watanabe et al. (2003) tested 15 new halogenated prostaglandins isolated from the Okinawan soft coral *Clavularia viridis.* Among these prostaglandins, three belong to iodovulone; seven belong to 12-*O*-acetyliodovulones, 12-*O*-acetylbromovulones, and 12-*O*-acetylchlorovulones; and the rest belong to 10,11-epoxy congeners of iodovulone, bromovulone, and chlorovulone. A simple compound, *p*-hydroxybenzaldehyde, was obtained from crude extracts of *Sinularia dissecta* (Jin, 2005) and *Muriceides collaris* ^※^ (Zhu et al., 2013). In addition, some simple aldehydes were isolated from *Antipathes dichotoma* Pallas^※^ (Ge et al., 2010), *Sinularia notanda* (Xu et al., 2017), *Scleronephthya* sp. (Huo et al., 2011), *D. gemmacea* ^※^ (*valenciennes*; Liu (2008)), *Dendronephthya* sp. (Li, 2004), and *Hicksonella guishanensis* Zou^※^ (Yu et al., 2004). *p*-Hydroxybenzoic acid can be extracted from *Subergorgia reticulata* (Xie et al., 2013) and red coral (Lai, 2017). Zou (2015) sorted out olefins from the crude extract of *Sinularia* sp. Subsequently, Li R. (2012) extracted ketones and alcohols from this coral. Esters such as methyl arachidonic acid (Liang et al., 2017), dibutyl phthalate, diisobutyl (Wang et al., 2009), and 1,2-benzenedicarboxylate (Lv et al., 2012) are also found in this coral.

## 4 Pharmacological effects and mechanisms

Many structurally active unique secondary metabolites, such as terpenoids, steroids, ceramides, and prostaglandins, have been extracted from corals, and their significant pharmacological activities, such as cytotoxic and antiviral activities, have been widely noticed and studied by natural product chemists and other researchers. Meanwhile, the pharmacological activities of coral bone powder and various coral preparations in the cardiovascular system have been explored. This article focuses on their cytotoxic effects on a variety of tumor cells and cancer cells as well as their restorative effects on bone injury diseases and their biological activities, such as antioxidant, anti-inflammatory, analgesic, and antiviral activities, on tissues of the nervous system and respiratory system. Accumulating evidence suggests that they have significant therapeutic effects on diseases of the nervous system.

### 4.1 Bone repair effect

The key to the treatment of bone defects is the suitability of the repair material. Autologous bone grafts cannot meet clinical needs for various reasons, and allogeneic and xenogeneic bones are limited in clinical application because of their antigenic nature (Zhou et al., 1993). The microstructure of coral and skeleton is also very close, specifically in its internal structure. Corals are divided into pinnate, laminate, branching and pith-like structures depending on the arrangement of the calcification centers. According to the skeleton body, the tiny tube traffic is divided into interlocking and interoperable traffic. Depending on whether the microscopic tubes in the skeleton are in traffic or not, they are divided into interlocking and interoperable. The interconnected coral skeleton has longitudinally and horizontally arranged tiny tubes, with pore diameters of 0.05–2.0 mm. Regardless of the section, these pores are interconnected. The coral artificial bone is widely valued as a promising material for bone repair (Roux et al., 1988). Animal experiments have shown that artificial bones made from horned honeycomb coral (favites) have good biocompatibility and osteocompatibility. When it is implanted in the mandible, the femoral cortical defect site after 8 months can be repaired, resulting in complete restoration (Zeng et al., 1997). Zhou et al. (1993) used Hainan Cheng Huang Bin Coral (Hainan Coral, Porites Iutea; HNC) composite implant material as the material graft in the side mandibular defect model and affirmed that the coral group led to the formation of new bone tissue, wrapped with phenanthrene fibrous tissue, followed by its better bone repair effect when used with BMG. In addition, it led to osseous healing, bone marrow cavity formation, and clear visualization of new bone tissue. Lai (2017) concluded that red coral can promote fracture healing and reduce the fracture healing period. According to the literature (Souyris et al., 1985; Dagli et al., 1997; Zhou, 2014), coral can also be used to correct saddle nose deformities, oral implants, skull injuries or postoperative repairs, and other orthopedic disorders.

In addition, coral transplants in the human body do not cause rejection; countless fine pores in the coral facilitate the gradual growth of microscopic blood vessels and synthesis of living cells of bone (Ma, 1994). Guillemin et al. showed that the resorption of corals starts with the growth of granulation tissue and blood vessels from the bone marrow into the coral. Then, the coral is progressively resorbed by many osteoclasts near its edges, while the woven bone formed with osteoclasts gradually grows into the resorbed void; finally, bone marrow cavity is formed, and the newly formed bone tissue system is clear and visible (Guillemin et al., 1989; Lu and Chen, 1994).

Chemical compounds extracted from corals also play a role in bone injury diseases. Lin Y. F. et al. (2013) isolated Ya-s11 (9 mg/kg) from the Taiwanese soft coral *Sinularia querciformis*, which not only attenuated AIA-induced ankle joint pathological changes but also significantly reduced the expression of osteoclast-related proteins.

### 4.2 Cytotoxicity

As shown in Table 2, studies in the literature in the last two decades have found that compounds extracted from coral have good cytotoxicity, particularly diterpenes, sesquiterpenes, sterols, and a small number of alkaloids, prostaglandins, and esters as active substances that also have some biological activity. These compounds are mostly extracted from corals of the genera *Sinularia*, *Lobophytum*, and *Sarcophyton* all belonging to the family Alcyoniidae. Corals of the family Gorgoniidae^※^ are also used as a source of active natural substances. The evaluation of their cytotoxic activity against tumor cells such as A549, HL-60, MCF-7, colon cancer cells, K562, and HeLa, followed by HepG2, Hep3B, MDA-MB-231, P-388, HT-29, MCF-7, Sup-T1, U937 and other cells, has become the hotspots of research. Shaaban et al. (2021) evaluated the *in vitro* anticancer effects of hydroazulenes, an extract of the soft coral *S. glaucum*, on colon (Caco-2) and breast (MCF-7) cell lines by MTT assays and showed that its antiproliferative or antiangiogenic effects were ultimately achieved by inhibiting the migration of MCF-7 cells and significant inactivation of VEGFR2 enzymes. Interestingly, the growth inhibitory concentrations of 5α-3β,6α,11-trihydroxy-24-methyl-9,11-seco-5a-cholest-7-en-9-one on colon (Caco-2) and breast (MCF-7) cell lines were 0.62 and 2.3 mM, respectively, but no toxicity was recorded against RPE-1 cells at a high concentration of 10 mM. The team also studied for the first time the anticancer properties of the sterol 10-epicatechin methyl ether. The first study of *Sarcophyton acutum* extract activity by Sabry A. H. Zidan studied for the first time the cytotoxic activity of *Sarcophyton acutum* extract and showed that polyhydroxylated steroid compounds had significant cytotoxicity to the HepG2 cell line (semi-inhibitory concentration 17.2 ± 1.5 μg/mL) and MCF-7 (semi-inhibitory concentration 33.2 and 25.1 mM) (Zidan et al., 2020; Abdelkarem et al., 2021) and that the side chains of polyhydroxylated sterols play an important role in the cytotoxic activity of such sterols. The researchers also demonstrated using the SRB method that the gorgonian of *Euplexaura rhipidalis* ^※^ has a significant apoptosis-inducing effect on A549 and HepG2 cells (Gong et al., 2017); in other words, prostaglandins with hydroxyl and carboxylic acids possess good cytotoxic properties and that they may have potential inhibitory effects on certain types of cancer (Hurtado et al., 2020). In fact, more than a decade ago, studies showed that the structure of compounds could influence cytotoxicity. A free hydroxyl group at C-12 or C-22 is important for enhancing the cytotoxic activity of a sterol against HeLa cell lines. In addition, the introduction of hydroxyl groups at C-20 decreased the inhibitory potency against HeLa cell lines, while the presence of acetoxy groups at C-18 seemed to enhance the cytotoxic activity (Zhang J. et al., 2013).

**TABLE 2:** Classification statistics for cytotoxicity of active substances extracted from the coral.

Active ingredient	Source	Activity	Concentration*	Target cell	Reference
Sesquiterpenoids	*Muriceides collaris* ^※^	—	50 μg/mL (Y)	P388 and BEL-7402	Shi (2009)
Sesquiterpenoids	*Litophyton arboreum*	—	4.32 ± 0.13–44.52 ± 0.5 μM (IC_50_)	MCF-7	Abou El-Kassem et al. (2018)
Sesquiterpenoids	*Xenia* sp.	—	5.89–6.45 μM (IC_50_)	STI	Phan et al. (2019)
Sesquiterpenoids	*Lemnalia* sp.	—	15.9 μM (IC_50_)	CCRF-CEM	Yan et al. (2021)
Sesquiterpenoids	*Sarcophyton glaucum*	—	18.8 ± 0.07, 19.9 ± 0.02 (HEPG2), 9.9 ± 0.03, 2.4 ± 0.04, 3.2 ± 0.02 (MCF-7), 29.4 ± 0.03, 19.4 ± 0.02, and 25.8 ± 0.03 (HCT116) μM (IC_50_)	HepG2, MCF-7, and HCT116	Abdel-Lateff et al. (2015)
Sesquiterpenoids	*Muriceides collaris* ^※^	—	50 μmol/L (Y)	HL-60 and HeLa	Zhu et al. (2013)
Sesquiterpenoids	*Sinularia kavarattiensis*	Antiproliferation	17.5 and 16.8 μM (IC_50_)	Leukemia and prostate cancer	Rajaram et al. (2013)
Sesquiterpenoids	*Sinularia scabra*	—	9.6–13.8 μg/mL (ED_50_)	MCF-7, WiDr, Daoy, and HEp-2	Su et al. (2012)
Sesquiterpenoids	*Sinularia* cf. *molesta*	—	5.26 and 8.37 μM (IC_50_)	HeLa and HCT116	Chu et al. (2018)
Sesquiterpenoids	*Sinularia* sp.	Cells that inhibit apoptotic proteins and trigger apoptosis by regulating Nrf2-ARE signaling	61.22 and 43.73 μM (Y)	HCT116	Taira et al. (2018)
Sesquiterpenoids and Steroids	*A. ochracea*	—	3.70–29.03 μg/mL (IC_50_)	HepG2, Hep3B, MCF-7/ADR, PC-3, HT-116, and Caski	Sun (2012)
Sesquiterpenoids and lactone	*Melithaea* sp.	—	50 μg/mL (Y)	K562, P388, and HeLa	Su (2011)
Diterpene	*Nephthea* sp.	—	37 μg/mL (IC_50_)	MCF-7	Hegazy et al. (2016)
Diterpene	*Lobophytum* sp.	—	4.52–6.62 μM (IC_50_)	HT-29, Capan-1, A549, and SNU-398	Li et al. (2020b)
Diterpene	*Sinularia flexibilis*	—	6.9–26.7 μM (IC_50_)	P-388, K-562, and HT-29	Wu et al. (2018)
Diterpene	*Lobophytum* sp.	—	1.8–8.2 μM (IC_50_)	A549 and HT-29	Nguyen et al. (2010)
Diterpene	*Cladiella* sp.	—	4.7and 10.2 μM (IC_50_)	CCRF-CEM	Chen et al. (2010b)
Diterpene	*Cladiella* sp.	—	2.0–31.1 μg/mL (IC_50_)	DLD-1 and HL-60	Chen et al. (2011b)
Diterpene	*Lobophytum laevigatum*	Inhibition of transcriptional activity	9.0 ± 0.8–38.8 ± 3.8 μM (IC_50_)	HL-60, A549, HCT116, and MCF-7	Quang et al. (2011a)
Diterpene	*Asterospicularia laurae*	—	1.3–19.41 μM (IC_50_)	Molt 4, K562, Sup-T1, and U937	Su et al. (2021)
Diterpene	*Dichotella gemmacea* ^※^	—	11.4–72.0 μM (IC_50_)	A549 and MG63	Li et al. (2016)
Diterpene	*Cladiella krempfi*	—	8.5 ± 1.0–18.1 ± 1.5 μg/mL (ED_50_)	H1299 and BT483	Tai et al. (2011)
Diterpene	*Sinularia triangular*	Antiproliferation	26.0–37.1 μM (ED_50_)	CCRF-CEM and DLD-1	Su, J.H. et al. (2011)
Diterpene	*Cespitularia taeniata*	—	0.3, 6.7, and 8.7 μM (IC_50_)	Medulloblastoma and colon adenocarcinoma cancer cells	Lin et al. (2014)
Diterpene	*Sinularia gibberosa*	Anti-invasion and anti-metastasis	4–8 μM (Y)	HA22T, RT4, and T24 human bladder cancer and HCC	Wu et al. (2020c)
Diterpene	*Nephthea* sp.	—	25, 70, 40, and 125 μg/mL (IC_50_)	HeLa/MCF-7	Ishii et al. (2016)
Diterpene	*Lobophytum* sp.	—	5.99–10.83 μM (IC_50_)	HeLa, A459, B16-F10, and RAW 264.7	Roy et al. (2019)
Diterpene	*Klyxum flaccidum*	—	16.5–49.4 μM (IC_50_)	HT-29, A549, K562, and P388	Ahmed et al. (2017a)
Diterpene	*Lobophytum crassum*	—	1.2–2.5 μg/mL (IC_50_)	Ca9-22	Chao et al. (2008a)
Diterpene	*Sinularia humilis*	—	12.5 μM (IC_50_)	HT-29	Li et al. (2022a)
Diterpene	*Lobophytum* sp.	—	1.2–8.6 μg/mL (IC_50_)	SGC7901, A549, MCF-7, HCT116, and B16	Zhao et al. (2013a)
Diterpene	*Sarcophyton elegans*	—	10 μM (Y)	MDA-MB-231	Liu et al. (2015)
Diterpene	*Sinularia microclavata*	—	5.0, 20.0 (KB, MCF), and 0.5 (A-549) μg/mL (IC_50_)	KB, MCF, and A-549	Zhang et al. (2005a)
Diterpene	*Lobophytum michaelae*	—	0.3–61.5 μM/mL (ED_50_)	HT-29 and P-388	Wang and Duh (2012)
Diterpene	*Nephthea* sp. *and Sarcophyton cherbonnieri*	Apoptosis	0.15–8.6 μg/mL (GI_50_)	HM02, HepG2, and MCF-7	Gross et al. (2003)
Diterpene	*Sinularia flexibilis*	—	0.16–32.4 μg/mL (ED_50_)	A549, HT-29, KB, and P-388	Duh et al. (1998)
Diterpene	*Pseudopterogorgia acerosa* ^※^	—	1.25–>8.10 μM (GI_50_)	DU-145, LNCaP, IGROV, IGROV-ET, SK-BR-3, SK-MEL-28, A549, PANC1, HT29, HT29-KF, LoVo, LoVo-DOX, HeLa, and HeLa-APL	Montalvo et al. (2006)
Diterpene	*Sinularia gibberosa*	—	18.7, 19.5, and 11.0 μg/mL (IC_50_)	HepG2 and A549	Chen et al. (2009)
Diterpene	*Sinularia flexibilis*	—	0.7–16.0 μg/mL (ED_50_)	KB, A-549, HT-29, and P388	Hsieh et al. (2003)
Diterpene	*Clavularia inflata*	—	0.052–27.3 μg/mL (ED_50_)	A549, HT-29, and P-388	Duh et al. (2001)
Diterpene	*Lobophytum* sp.	Apoptosis	3.7 (HT-29), 5.1 (A549), and 6.6 (SNU-C5) μM (IC_50_)	HT-29, A549, and SNU-C5	Hong et al. (2012)
Diterpene	*Sinularia* sp.	—	7.98–17.23 μM (IC_50_)	HCT116	Xu (2013)
Diterpene	*Dichotella gemmacea* ^※^	—	3.8–112.3 μg/mL (IC_50_)	A549 and MG63	Jiang (2013)
Diterpene	*Sarcophyton latum*	—	50 μg/mL (Y)	P388, A549, and BEL-7402	Wang (2008)
Diterpene	*Sinularia dura*	Antiproliferation and anti-invasion	20–30	Highly malignant + - SA breast epithelial cells, PC-3 M-CT+	Radwan et al. (2008)
Diterpene	*Sarcophyton trocheliophorum*	—	10 μmol/L (Y)	A-549 and HL-60	He (2013)
Diterpene	*Lobophytum* sp.	—	1.83–44.69 μg/mL (IC_50_)	B16F10, HeLa, and HepG2	Lang (2013)
Diterpene	*Lobophytum* sp.	—	50 μg/mL (Y)	P388 and HeLa	Fernando et al. (2017)
Diterpene	*Cladiella krempfi.*	—	6.7 ± 0.7–19.2 ± 4.0 μg/mL (IC_50_)	A549, BT483, H1299, HepG2, and SAS	Tai et al. (2013)
Diterpene	*Sinularia* sp.	Apoptosis	—	HL-60	Kamada et al. (2018)
Diterpene	*Sinularia* sp.	—	0.0039 μg/mL	HL-60, PC-3MIE8, and BGC-823	Li (2004)
Diterpene	*Dichotella gemmacea* ^※^	—	10.6–70.0 μM (IC_50_)	A549, HL-60, and K562	Sun (2012)
Diterpene	*Cladiella*	Directly affecting tumor growth and angiogenesis	1.6 (MDA-MB-231 cell)/>10 μM (IC_50_)	EGF-dependent cancers	Mohyeldin et al. (2017)
Diterpene	*Sarcophyton mililatensis*	—	0.78–1.26 μM (IC_50_)	HL-60 and A549	Li (2018)
Diterpene	*Clavularia* sp.	—	50 μM (Y)	K562, HL-60, HeLa, and A549	Xue (2014)
Diterpene	*Sinularia* sp.	—	2.32–8.97 μM (IC_50_)	K563	Zou (2015)
Diterpene	*Anthoptilum grandiflorum*	Killed the NT2 cells and antiproliferation	—	NT2	Thomas et al. (2019)
Diterpene	*Sarcophyton crassocaule*	—	2.0, 1.2, 2.6, and 3.2 μM (ED_50_)	MCF-7, WiDr, HEp-2, and Daoy cancer cell lines	Lin et al. (2010)
Diterpene	*Briareum* sp.	Reduced the expression of COX-2	5–30 μM (IC_50_)	Caco-2 cells	Joyner et al. (2011)
Diterpene	*Dichotella gemmacea* ^※^	Antiproliferation	5.0–78.5 μM (IC_50_)	A-549 and MG63	Li et al. (2013a)
Diterpene	*Pseudopterogorgia kallos* ^※^	—	<0.01, 0.51 μM (GI_50_)	EKVX non-small-cell lung cancer and Caki-1 renal cancer	Marrero et al. (2004)
Diterpene	*Lobophytum crassum*	Inhibition of transcriptional activity	6.30 ± 0.42–6.63 ± 0.11 μM (IC_50_)	HepG2	Thao et al. (2014a)
Xenicane	*Protodendron repens*	—	0.2–6.3 μM (GI_50_)	MDAMB-231, HT-29, and NSLC A-549	Urda et al. (2017)
Terpenoids	*Sarcophyton* sp.	—	6.4–33.7 μM (IC_50_)	P338, A549, HL-60, and K562	Gong (2014)
Terpenoids	*Sarcophyton tortuosum*	—	3.5–24.7 μg/mL (IC_50_)	Human nasopharyngeal carcinoma CNE-2 cell line and P-388	Zeng et al. (2004)
Terpenoids	*Sinularia* sp.	Inhibitory activity	6.5–33 μM (IC_50_)	E3-ubiquitin ligase casitas B-lineage lymphoma proto-oncogene B (Cbl-b)	Jiang et al. (2021)
Terpenoids	*Sarcophyton* sp.	—	6.03 ± 1.93, 6.70 ± 1.06 μM (IC_50_)	Canpan-1	Lu (2020)
Diterpene and steroids	*Sinularia dissecta*	—	2.54–100 μg/mL (IC_50_)	PC-3MIE8 and A549	Jin (2005)
Diterpene and steroids	*Lobophytum compactum*	—	17.80 ± 1.43–59.06 ± 2.31 μM (IC_50_)	A549 and HL-60	Chau et al. (2011)
Diterpenoid lactone and steroids	*Sinularia polydactyla*	—	1.0, 6.1, and 8.2 μg/mL (IC_50_)	HepG2, HEp2, and HCT	Aboutabl el et al. (2013)
Steroids	*Sinularia gibberosa*	Antiproliferation	6.8–10.0 μM (ED_50_)	Hepa59T/VGH	Ahmed et al. (2003)
Steroids	*Sarcophyton glaucum*	Antiproliferation	0.62 and 2.3 μM (IC_50_)	Caco-2 and MCF-7	Shaaban et al. (2021)
Steroids	*Sinularia erecta*	—	15.57 ± 5.26–40.55 ± 7.51 μM (IC_50_)	A549, HT-29, SNU-398, and Capan-1	Liu et al. (2020)
Steroids	*Verrucella corona*	—	12.32 ± 1.47–33.77 ± 1.28 μM (IC_50_)	LNCaP, HepG2, KB, MCF-7, SK-Mel2, HL-60, LU-1, and SW480	Nam et al. (2018)
Steroids	*Sinularia leptoclados*	—	13.45 ± 1.81–29.01 ± 3.21 μM (IC_50_)	HepG2, SW480, HL-60, MCF-7 LU-1, SK-Mel2, and LNCaP	Ngoc et al. (2017b)
Steroids	*Heteroxenia fuscescens*	—	33.2 and 25.1 μM (IC_50_)	MCF-7	Abdelkarem et al. (2021)
Steroids	*Nephthea erecta*	Apoptosis and increases caspases activity	20 and 40 μM (Y)	H1688 and H146 lung cancer	Chung et al. (2017a)
Steroids	*Sinularia suberosa*	—	5.5–6.5 μM (IC_50_)	K562 and MDA-MB-231	Zhang (2013a)
Steroids	—	—	21.56–40.04 μM (IC_50_)	HT-29, SNU-398, and Capan-1	Zhang (2019)
Steroids	*Rumphella aggregata* ^※^	—	10 μg/mL (Y)	K562	Liu et al. (2012)
Steroids	*Nephthea* sp.	—	7.51 ± 0.22–18.72 ± 0.78 μg/mL (IC_50_)	HeLa	Zhang et al. (2013b)
Steroids	*Pacifigorgia senta* ^※^	—	7.0–29.7 μM (IC_50_)	HepG2, Hep3B, MCF-7/ADR, PC-3, and HCT116	Chen et al. (2016)
Steroids	*Paragorgia* sp.	Antiproliferation	3.0–90 μM (GI_50_)	A-549, HT-29, and MDA-MB 231	Poza et al. (2008)
Steroids	*Clavularia viridis*	—	0.1–6.8 μg/mL (IC_50_)	HT-29 and P-388	Duh et al. (2007)
Steroids	*Stereonephthya crystalliana*	—	1.6–13.3 μg/mL (ED_50_)	HT-29 and P-388	Wang et al. (2006)
Steroids	*Sinularia* sp.	—	0.69, 4.03, and 1.79 μM (IC_50_)	HL-60	Li et al. (2018a)
Steroids	*Menella kanisa* ^※^	Antiproliferation	11.0 ± 4.2–257.2 ± 20.7 μM (IC_50_)	A549 and MG-63	Wang, P. et al. (2013)
Steroids	*Subergorgia suberosa*	—	15.1 μM (IC_50_)	HeLa	Zhang et al. (2015a)
Steroids	*Sinularia polydactyla*	Anti-migration and neuroprotective activity on nerve cells	10,20	HeLa, MCF-7, and SH-SY5Y	Tammam et al. (2020)
Steroids	*Sinularia brassica*	—	1.17 ± 0.42–92.53 ± 1.68 μM (IC_50_)	A-549, HeLa, and PANC-1	Tran et al. (2017)
Steroids	*Scleronephthya gracillimum*	—	23.3, 21.9, and 24.3 μM (IC_50_)	HepG2, A549, and MDA-MB-231	Fang et al. (2013)
Steroids	*Carijoa* sp.	—	9.33, 11.02, and 18.68 μM (IC_50_)	Bel-7402	Zhao et al. (2013c)
Steroids	*Sarcophyton* sp.	—	6.4–10.3 μM (IC_50_)	HL-60, HeLa, and K562	Gong et al. (2013)
Steroids	*Sinularia* sp.	—	8.36–37.30 μM (IC_50_)	HepG2 and HeLa	Sun et al. (2016)
Steroids	*Sarcophyton* sp.	—	5.25, 12.30, 4.95, 4.10 (K562), 7.30, and 6.20 (A549) μg/mL (IC_50_)	K562 and A549	Sun et al. (2013)
Steroids	*Subergorgia suberosa*	Inhibiting activity	5.5, 6.2, and 6.5 μM (IC_50_)	K562 and MDA-MB-231	Zhang et al. (2013a)
Steroids	*Klyxum flaccidum*	—	12.7–15.5 μM (IC_50_)	HT-29, P388, and K562	Tseng et al. (2016)
Steroids	*Nephthea chabrolii*	—	1.1, 1.2, and 1.0 μg/mL (ED_50_)	P-388, A-549, and HT-29	Shang-Kwei et al. (2013)
Steroids	*Lobophytum laevigatum*	Apoptosis and antiproliferation	3.2–18.1 μM (IC_50_)	HCT-116, A549, and HL-60	Quang et al. (2011b)
Steroids	*Nephthea* sp.	—	2.3*(10^−7^)–98.5*(10^−4^)	HL-60 and A-549	Ma (2008)
Steroids	*Lobophytum* sp.	—	21.56–38.83 and 40.04 μM (IC_50_)	HT-29, SNU-398, and Capan-1	Zhang et al. (2019)
Steroids	*Litophyton mollis*	—	10 μM (IC_50_)	K562 and PBMCs	Zovko Končić et al. (2016)
Steroids	*Nephthea erecta*	—	6.5–14.0 μM (IC_50_)	K562, Molt-4, Sup-T1, and U937	Tsai et al. (2016)
Steroids	*Lobophytum michaelae*	—	14.9 ± 5.7 μg/mL (IC_50_)	A549	Huang et al. (2018)
Steroids	*Verrucella corona*	—	12.32 ± 1.47–33.77 ± 1.28 μM (IC_50_)	LNCaP, HepG2, KB, MCF-7, SK-Mel2, HL-60, LU-1, and SW480	Nam et al. (2018)
Steroids	*Sinularia microspiculata*	—	72.32 ± 1.30–89.02 ± 9.93 μM (IC_50_)	HL-60 and SK-Mel2	Thanh et al. (2016)
Steroids	*Sarcophyton acutum*	—	17.2 ± 1.5 and 24.8 ± 2.8–57.2 ± 5.2 μg/mL (IC_50_)	HepG2, MCF-7, and A549	Zidan et al. (2020)
Steroids		Ability to induce autophagy	20 μM (Y)	MCF-7	Weng et al. (2018)
Steroids	*Cladiella hirsuta*	—	8.2–42.0 μM (IC_50_)	HepG2, HepG3B, MDA-MB-23, and Ca9-22	Chen et al. (2011a)
Steroids	*Sinularia variabilis*	Apoptosis	—	MCF-7 and MDA-MB-231	Mohammadi Pour et al. (2022)
Steroids	*Spongodes* sp.	—	0.14, 5, and 3.8 μg/mL (IC_50_)	BEL-7402, A-549, HT-29, and P388	Yan et al. (2007)
Steroids	*Sinularia acuta*	—	7.28–44.82 μM (IC_50_)	HL-60, K562, and HeLa	Zhang (2014)
Steroids	*Carijoa* sp.	—	9.33–18.68 μM (IC_50_)	Bel-7404	Zhao (2013)
Steroids	*Sarcophyton* sp.	—	—	K562	Sun (2012)
Steroids	*Sinularia* sp.	—	1.79 and 4.03 μM (IC_50_)	HL-60	Li (2018)
Steroids	*Sinularia* sp.	Antiproliferation	1.61 and 3.26 μmol/L (IC_50_)	HL-60	Li et al. (2018b)
Steroids	*Sinularia* sp.	Apoptosis	10.14–41.71 μM (IC_50_)	MDA-MB-436, A549, Hep3B, HT-29 and H157	Jiang et al. (2019b)
Steroids	*Subergorgia suberosa*	—	1.09–6.22 μM (IC_50_)	K562	Liu (2014)
Steroids and ceramide	*Cespitularia stolonifera*	—	23.0–1,574.0 μg/mL (IC_50_)	A549 and MCF-7	Elshamy et al. (2017)
Alkaloid	*Ellisella robusta* ^※^	—	0.35–58.01 μM (IC_50_)	HeLa and K562	Zhang (2012b)
	*Ellisella curvata* ^※^				
Alkaloid	*Menella kanisa* ^※^	Inhibiting activity and antiproliferation	13.3, 55.0 μg/mol (IC_50_)	Osteosarcoma cells	Yao et al. (2015)
Alkaloid	*Muriceides collaris* ^※^	—	5.08–8.37 μM (IC_50_)	K562 and HeLa	Zhu (2013)
Alkaloid	*Scleronephthya* sp.	Anti-metastasis	5.3 ± 0.2–12.4 ± 0.2 μM (IC_50_)	A549 and B16	Cheng et al. (2017)
Prostanoids	*Clavularia viridis*	Apoptosis	0.12–11.7 μM (IC_50_)	Prostate cancer PC-3 cells	Chiang et al. (2006)
Prostanoids	*Clavularia viridis*	Antiproliferation	0.5–7.9 μM (IC_50_)	PC-3 and HT29	Shen et al. (2004)
Prostanoids	*Plexaura homomalla* ^※^	Inhibiting the expression of related enzymes	16.46, 25.20 μg/mol (IC_50_)	MDA-MB-213 and A549	Hurtado et al. (2020)
Ester	*Sinularia flexibilis*	Antiproliferation	10 mg/kg (Y)	Small cell lung cancer	Lin et al. (2013a)
Ester	*Cladiella kashmani*	Anti-invasion and anti-metastasis	1, 2.5, 5, and 10 μM (Y)	T24 human bladder cancer cells	Wu et al. (2019a)
Ester	*Paraminabea acronocephala*	—	0.5–2.2 μM (IC_50_)	HepG2, Hep3B, MDA-MB-231, MCF-7, and A-549	Chao et al. (2011b)
Ester	*Lobophytum durum*	—	3.8 μg/mL (ED_50_)	P-388	Cheng et al. (2011)
Ester	*Sinularia flexibilis*	Anti-invasion and anti-metastasis	—	Gastric cancer	Wu et al. (2019b)
Ester	*Stragulum bicolor*	Apoptosis	0.18 and 4.3 μM (IC_50_)	A2058	Nuzzo et al. (2019)
Sinulariolide	*Sinularia flexibilis*	Antiproliferation and apoptosis	15 μM (Y)	Bladder carcinoma cell and TSGH cells	Neoh et al. (2012)
Alkane	*Montipora* sp.	—	1.40–29.16 μg/mL (ED_50_)	A549, SK-OV-3, SK-MEL-2, XF498, and HCT15	Alam et al. (2001)
Aromatic compounds	*Scleronephthya gracillimum*	—	2.86–7.51 μg/mL μM (IC_50_)	HeLa and P388	Han (2011)
				HepG2, Hep3B, and HT116	
Oligopeptides	*Sarcophyton glaucum*	—	8.6, 4.9, and 5.6 mmol/L (EC_50_)	HeLa	Quah et al. (2019)
EPA	*Eunicea succinea* ^※^	—	5.1–6.9 μmol/L (IC_50_)	Malignant glioma U87-MG and U373-MG cells	Iwamaru et al. (2007)
Lobophorin	*Lophelia pertusa*	—	6.3 ± 8.2, 23.0 ± 8.9, and 34.0 ± 85.1 μM (IC_50_)	MiaPaca-2, MCF-7, and THLE-2	Braña et al. (2017)
Tetraphenylbenzoquinone	*Sinularia capillosa*	—	9.8 and 12.7 μM (ED_50_)	P-388	Cheng et al. (2010a)
Durumolide	*Sinularia polydactyla*	—	1.0–8.2 μg/mL (IC_50_)	HepG2, HEp2, and HCT	Aboutabl el et al. (2013)
Biscembranoids	*Sarcophyton pauciplicatum*	—	7.93 ± 2.08–94.18 ± 3.02 μM (IC_50_)	LNCaP MCF-7 KB HepG2, SK-Mel2, HL-60, SW480, and LU-1	Nam et al. (2015)
Tryptamine derivatives	*Eunicella granulata* ^※^	—	1.7–12.7 μM (GI_50_)	DU-145, LNCaP, SK-OV-3, IGROV, IGROV-ET, SK-BR3, SK-MEL-28, A549, K-562, PANC1, HT29, LoVo LoVo-DOX, HeLa, and HeLa-APL	Reyes et al. (2006)
Tetracyclic biscembranes	*Sarcophyton glaucum*	—	13.3–58.0 μM (IC_50_)	HL-60	Iwagawa et al. (2009)
Sinularin	*Sinularia flexibilis*	Increasing G2/M cell cycle arrest, inducing apoptosis, and activating DNA damage responses	17.5 ± 6.7, 9.4 ± 2.3 (HEPG2), 43.2 ± 8.1, and 33.9 ± 8.6 μM (Hep3B) μM (IC_50_)	HepG2 and Hep3B	Chung et al. (2017b)
13-Acetoxysarcocrassolide	*Sarcophyton crassocaule*	Apoptosis	1 and 1.5 μg/mL (Y)	BFTC	Su, C.C. et al. (2011)
Flaccidoxide-13-acetate	*Sinularia gibberosa*	Apoptosis	20 μM (Y)	RT4 and T24 human bladder cancer cells	Wu et al. (2019a)
Glycolipids	*Lobophytum crassum*	—	9.2–15.0 μg/mL (IC_50_)	HepG2, Hep3B, MDA-MB-231, and Ca9-22	Chao et al. (2007)
Crude extract	*Sinularia* cf*. molesta*	—	50 μg/mL (Y)	K562 and HL-60	Jiang (2015)
—	*Muricella sibogae*	—	1, 10, and 50 μg/mL (Y)	P388 and BEL-7402	Li (2010b)
—	*Cladiella australis*, *Clavularia viridis*, *and Klyxum simplex*	Apoptosis	31.5 ± 1.5–53.8 ± 2.1 μg/mL (IC_50_)	Squamous cell carcinoma cells	Liang et al. (2008)
—	*Carotalcyon* sp.^※^	Antiproliferation and apoptosis	0.7 ± 0.4–250.9 ± 92.1 μg/mL (IC_50_)	HGUE-C-1, HT-29, and SW-480	Ruiz-Torres et al. (2019)
—	*Euplexaura rhipidalis* ^※^	Apoptosis	<10 μg/mL (IC_50_)	A549 and HepG2	Gong et al. (2017)
—	*Sinularia maxima*	Inhibition of transcriptional activity	15.81 ± 2.29–29.10 ± 1.54 μM (IC_50_)	HepG2	Thao et al. (2014b)

*Y refers to the medication.

### 4.3 Anti-inflammatory and analgesic effects

Inflammatory processes usually constitute the initial activation of the mammalian immune system and the body’s normal defense or protective mechanisms against microbial infections or stimuli, tissue, or organ damage. Accumulating evidence shows a critical link between inflammation and the chronic promotion/progression of various human diseases, including atherosclerosis, diabetes, arthritis, inflammatory bowel disease, cancer, and Alzheimer’s disease (Wei et al., 2013). Different types of cells, such as monocytes/macrophages, neutrophils, and lymphocytes, are involved in the inflammatory process (Serhan and Savill, 2005). Several marine biology and chemistry researchers have systematically screened the *in vitro* anti-inflammatory activity of several marine natural products isolated from corals, and lipopolysaccharide-stimulated mouse macrophage models have been widely used as a system for assessing the anti-inflammatory activity of secondary metabolites of marine and terrestrial origin (Lin et al., 2015). Yen-You Lin’s study showed that the diterpene compound excavatolide B from the gorgonian of *Briareum excavatum* ^※^ produced potent anti-inflammatory activity *in vitro* and *in vivo* and inhibited the expression of iNOS and COX-2 mRNA. Gyrosanols A and B show significant anti-inflammatory activity by reducing COX-2 protein levels in RAW 264.7 macrophages (Cheng et al., 2010a). Lee et al. (2013) found that soft coral-derived leminalol attenuated monosodium urate-induced gouty arthritis in rats by inhibiting leukocyte infiltration and the expression of iNOS and COX-2 proteins, among others.

The inflammatory process also involves the peripheral and central nervous system (CNS) and is thought to be involved in the pathogenesis of neuropathic pain (Ellis and Bennett, 2013s). Chen N. F. et al. (2014) investigated flexibilide, extracted from cultured soft corals, as a possible drug for neuropathic pain, and its anti-neuritis and analgesic mechanisms of action may be related to spinal TGF-β1 inhibition. The sphingosine derivative obtained from soft corals also has anti-inflammatory and analgesic effects (Radhika et al., 2005). After compiling nearly 100 studies, it was found that the anti-inflammatory activity of coral extracts is mainly attributed to diterpene compounds, followed by sterols, prostaglandins, and alkaloids. Its anti-inflammatory activity is mainly mediated by the inhibition of lipopolysaccharide-induced expression of iNOS and COX-2 in mouse macrophages (RAW 264.7) or by the inhibition of superoxide anion release from human neutrophils FMLP/CB and elastin. The specific functions of anti-inflammatory and analgesic effects in corals are shown in Table 3.

**TABLE 3:** Classification statistics for anti-inflammatory and analgesic effects of active substances extracted from the coral.

Active ingredient	Source	Activity	Concentration^a^	Reference
Sesquiterpenoids	*Sinularia tumulosa*	I	2.6–7.5 µM (IC_50_)	Cai et al. (2020)
Sesquiterpenoids	*Anthogorgia* sp.	N and A	27.81 μg/mL (IC_50_)	Ji and Liu (2018)
Sesquiterpenoids	*Sinularia scabra*	I	10 µM (Y)	Su et al. (2012)
Diterpene	*Lobophytum crassum*	I and C	10 µM (Y)	Chao et al. (2008a)
Diterpene	*Cladiella krempfi*	I and C	10 µM (Y)	Tai et al. (2013)
Diterpene	*Briareum* sp.	C	5–30 µM (Y)	Joyner et al. (2011)
Diterpene	*Lobophytum* sp.	N	5, 10, and 25 µM (Y)	Roy et al. (2019)
Diterpene	*Klyxum flaccidum*	N	50, 46.7, and 47.0 (IC_50_)	Ahmed et al. (2017b)
Diterpene	*Sinularia flexibilis*	S and E	10.8 ± 0.38 and 11.0 ± 1.52 µM (IC_50_)	Wu et al. (2018)
Diterpene	*Cladiella krempfi*	I	10 µM (Y)	Tai et al. (2011)
Diterpene	*Sinularia triangular*	I, C	10 µM (Y)	Su and Wen (2011)
Diterpene	*Lobophytum laevigatum*	I and C	0.1–10 Y	Quang et al. (2011a)
Diterpene	*Sarcophyton glaucum*	A	20 μmol/L (Y)	Li et al. (2022b)
Diterpene	*Sinularia flexibilis*	N	10 µM (Y)	Xu (2016)
Diterpene	*Briareum excavatum* ^※^	I and C	10 µM (Y)	Huynh et al. (2020)
Diterpene	*Briareum* sp.	I	10 µM (Y)	Su et al. (2015a)
Diterpene	*Briareum* sp.	I and C	10 µM (Y)	Su et al. (2015b)
Diterpene	*Lobophytum crassum*	N	2.4 ± 0.21–16.6_x0007_ 1.70 (IC_50_)	Wanzola et al. (2010)
Diterpene	*Lobophytum varium*	S and E	10 µM (Y)	Ahmed et al. (2017a)
Diterpene	*Lobophytum crassum*	N	50 μg/mL (Y)	Chao et al. (2008b)
Diterpene	*Sinularia gyrosa*	C	10 µM (Y)	Cheng et al. (2010a)
Diterpene	*Lobophytum durum*	I and C	10 µM (Y)	Cheng et al. (2009a)
Diterpene	*Sinularia querciformis and Sinularia granosa*	I and C	10 µM (Y)	Lu et al. (2008)
Diterpene	*Cladiella* sp.	S and E	10 µM (Y)	Chen et al. (2010b)
Diterpene	*Cladiella* sp.	S and E	8.1 ± 0.3–49.4 ± 0.2 (IC_50_/Inh)	Chen et al. (2011b)
Diterpene	*Klyxum simplex*	I and C	10 µM (Y)	Chen et al. (2010a)
Diterpene	*Lobophytum* sp.	N	3.2–9.4 µM (IC_50_)	Zhao et al. (2013b)
Diterpene	*Sinularia gyrosa*	C	10 µM (Y)	Cheng et al. (2010b)
Diterpene	*Sarcophyton cherbonnieri*	S and E	30 µM (Y)	Peng et al. (2020)
Diterpene	*Lobophytum crassum*	I and C	6.30 ± 0.42–6.63 ± 0.11 µM (IC_50_)	Thao et al. (2014a)
Diterpene	*Sarcophyton glaucum*	A	10 µM (Y)	Shen et al. (2021)
Diterpene	*Junceella fragilis* ^※^	I	10 µM (Y)	Su et al. (2019)
Diterpene	*Nephthea columnaris*	I and C	9.80 μg/mL (IC_50_)	Hsiao et al. (2015)
Diterpene	*Lobophytum durum*	I	10 µM (Y)	Cheng et al. (2009b)
Diterpene	*Sinularia maxima*	I	4.35 ± 0.12–59.77 ± 2.34 µM (IC_50_)	Thao et al. (2012)
Diterpene	*Sinularia maxima*	I	0.1, 1.0, and 10 µM Y	Thao et al. (2014b)
Diterpene	*Lobophytum pauciflorum*	N	2.8 µM (IC_50_)	Yan et al. (2010)
Diterpene	*Sinularia crassa*	I and C	10 µM (Y)	Chao et al. (2011a)
Diterpene	*Lobophytum sarcophytoides*	N	7.1–32.1 µM (IC_50_)	Shen et al. (2019)
Diterpene	*Klyxum molle*	I and C	10 µM (Y)	Hsu et al. (2011)
Diterpene	*Sarcophyton ehrenbergi*	I	7.2–38.6 µM (IC_50_)	Li et al. (2020a)
Diterpene	*Briareum excavatum* ^※^	I and C	1–50 µM (Y)	Lin et al. (2015)
Diterpene	*Sinularia crassa and Lobophytum* sp.	—	10 mg/kg (Y)	Radhika et al. (2005)
Diterpene	*Sinularia nanolobata*	N and A	20 µM (Y)	Zeng et al. (2021)
Diterpene	*Cladiella* sp.	S and E	1.97 ± 2.44–41.08 ± 3.26 μg/mL (IC_50_)	Chen et al. (2012)
Cembranoid	*Sarcophyton crassocaule*	I and C	10 µM (Y)	Lin et al. (2010)
Cembranoid	*Sinularia* sp.	I	<6.25 μg/mL (Y)	Kamada et al. (2018)
Norditerpenoids	*Sinularia maxima*	I	5.30 ± 0.21–69.85 ± 4.11 µM (IC_50_)	Thao et al. (2013)
Norditerpenoids	*Sinularia numerosa*	I	10 µM (Y)	Yin et al. (2015)
Norditerpenoids	*Sinularia siaesensis*	A	20 µM (Y)	Chen et al. (2021)
Norditerpenoids	*Sinularia maxima*	I	23.52 ± 1.37 and 69.85 ± 4.11 µM (IC_50_)	Thao et al. (2013)
Norditerpenoids	*Sinularia* sp.	N and I	33 μg/mL (Y)	Hiroko et al. (2003)
Norditerpene	*Sinularia gyrosa*	I	10 µM (Y)	Cheng et al. (2010c)
Nanolobatolide	*Sinularia nanolobata*	I	10 µM (Y)	Tseng et al. (2009)
Diterpene and sesquiterpenoids	*Cespitularia* sp.	I, C, and N	100 µM (Y)	Lin et al. (2021)
Steroids	*Nephthea chabroli*	I, C	10 µM (Y)	Huang et al. (2008)
Steroids	*Sinularia crassa*	I, C	10 µM (Y)	Chao et al. (2012)
Steroids	*Klyxum flaccidum*	S, E	4.40 ± 0.19, 5.64 ± 0.41 (IC_50_)	Tseng et al. (2016)
Steroids	*Nephthea chabroli*	I and C	10 µM (Y)	Huang et al. (2008)
Steroids	*Scleronephthya gracillimum*	I and C	10 µM (Y)	Fang et al. (2013)
Steroids	*Clavularia viridis*	I and C	10 µM (Y)	Chang et al. (2008)
Steroids	*Dendronephthya griffini*	I and C	10 µM (Y)	Chao et al. (2008a)
Steroids	*Echinomuricea spinosa* ^※^	S and E	1.13 ± 0.55–95.54 ± 6.17 µM (IC_50_)	Chung et al. (2012)
Steroids	*Dendronephthya gigantea*	I, C, S, E, and N	4.33 ± 0.50 μg/mL (IC_50_)	Fernando et al. (2017)
Steroids	*Pinnigorgia* sp.^※^	I and C	10 µM (Y)	Su et al. (2016)
Crude extract	*Nephthea* sp*.*	C	33.72–46.75 μg/mL (IC_50_)	Abdelhafez et al. (2020)
Flexibilisquinone	*Sinularia flexibilis*	I and C	5–20 µM (Y)	Lin et al. (2013b)
Tocopherol-derived	*Cladiella hirsuta*	S and E	3.7 ± 0.3–4.1 ± 1.1 µM (IC_50_)	Chen et al. (2015)
EGFR	—	C and I	10 µM (Y)	Lin et al. (2013a)
Lemnalol	—	I and C	30 mg/kg (Y)	Lee et al. (2013)
Lemnalol	*Lemnalia cervicornis*	A	0.05–10 µg (Y)	Lin et al. (2011)
Lemnalol	*Lemnalia cervicornis*	I and C	15 mg/kg (Y)	Jean et al. (2008)
Quinones	*Sinularia flexibilis*	I and C	5–20 μM (Y)	Lin et al. (2013b)
Glycoside	*Pseudopterogorgia elisabethae* ^※^	—	1–4 µM (IC_50_)	Mayer et al. (1998)
Briarane	*Junceella fragilis* ^※^	E	10 μg/mL (Y)	Sheu et al. (2006)
Isosarcophine	*Sarcophyton cherbonnieri*	S and E	30 µM (Y)	Peng et al. (2021)
Tetraphenylbenzoquinone	*Sinularia capillosa*	I and C	10 µM (Y)	Cheng et al. (2010a)
Withanolide	*Paraminabea acronocephala*	I and C	10 µM (Y)	Chao et al. (2011b)
Capnellene	*Capnella imbricate*	C	6.21 ± 2.5 and 17.9 ± 2.9 µM (IC_50_)	Jean et al. (2009)
Bicyclogermacrenes	*Capnella* sp.	I and N	10 and 20 µM (Y)	Phan et al. (2015)
Isoprenoids	*Sinularia erecta*	S and E	0.9 ± 0.1–8.5 ± 0.3 µM (IC_50_)	Lin et al. (2014)
Prostaglandin	*Plexaura homomalla* ^※^	V and E	100 µM (Y)	Huang et al. (2016)

^a^ Inhibition of iNOS (I), COX-2 (C), superoxide anion (S), N (N0), astrocytes (A), and elastase (E); Y refers to the medication.

### 4.4 Antiviral

Viruses are infectious entities that use the cellular biosynthetic machinery to replicate their own nucleic acids, synthesize the proteins encoded by their nucleic acids, and finally assemble into complete, infectious viral particles. In most cases, viruses can cause disease and even death in infected hosts (Li W. et al., 2022). Almost all clinical and public health outbreaks over the decades have been due to emerging viruses, including coronavirus (SARS), which causes severe acute respiratory distress syndrome, influenza A virus subtype H1N1 (IAV-H1N1), which caused an influenza pandemic in 2009, human cytomegalovirus (HCMV), which can cause visceral disease, and the SARS CoV-2, which caused a widespread outbreak worldwide in 2019 (Chen et al., 2023). The widespread outbreak of the virus not only poses a great threat to the lives and health of people across the country but also severely hinders global economic development. Marine organisms have been shown to be a rich source of antiviral drugs (Cao et al., 2014). Chun-Kuang demonstrated that lobohedleolide isolated from the Taiwanese soft coral *Lobophytum crassum* significantly reduced HCV replication in replicon cells and JFH-1-infected systems with EC_50_ values of 10 ± 0.56 and 22 ± 0.75 μM at nontoxic concentrations, respectively. Their study also concluded that the inhibitory effect on HCV replication was due to the inhibition of HCV-induced COX-2 expression (Lin et al., 2018). Gong et al. (2013) showed for the first time thatspecific types of steroids were active against influenza viruses. The antiviral effect of coral is mainly achieved through the inhibition of viral replication and expression of antigens. As summarized, coral mainly has antiviral activity against pathogens such as HCMV and H1N1, and some studies have also found antiviral activity against pathogens such as HBV and HCV, as shown in Table 4.

**TABLE 4:** Classification statistics for antiviral effects of active substances extracted from the coral.

Active ingredient	Source	Virus	Concentration^a^	Activity	Reference
Sesquiterpenoids	*Muriceides collaris* ^※^	H1N1	50 μM (Y)	—	Zhu (2013)
Sesquiterpenoids	*Lemnalia sp.*	H1N1	1.1 and 7.1 µM (IC_50_)	—	Liu et al. (2022)
Sesquiterpenoids	*Lemnalia sp.*	H1N1	5.9 µM (IC_50_)	—	Yan et al. (2021)
Sesquiterpenoids	*Echinogorgia flora* ^※^	H1N1	50 μM (Y)	—	Wu (2013)
Diterpene	*Sinularia gyrosa*	HCMV	2.6 and 3.7 μM (IC_50_)	—	Cheng et al. (2010b)
Diterpene	*Junceella fragilis* ^※^	HBeAg	0.89–6.47 μM (IC_50_)	Inhibition of HBeAg antigen expression	Wei et al. (2017)
Diterpene	*Ellisella sp.*	HBV and HBeAg	10 μM (Y)	Suppression of virus replication	Wu et al. (2020a)
Diterpene	*Clavularia sp.*	H1N1	50 μM (Y)	—	Xue (2014)
Diterpene	*Lobophytum durum*	HCMV	5.2 μg/mL (IC_50_)	Inhibition of viral transcription	Cheng et al. (2011)
Norditerpenoids	*Sinularia gyrosa*	HCMV	1.9 μg/mL (IC_50_)	—	Cheng et al. (2010c)
Steroids	*Echinogorgia rebekka* ^※^	Respiratory syncytial virus	0.19 μM (IC_50_)	—	Cao et al. (2014)
Steroids	*Sarcophyton sp.*	H1N1	19.6–36.7 μg/mL (IC_50_)	—	Gong (2014)
Steroids	*Sarcophyton sp.*	H1N1-IAV	19.6 and 36.7 μM (IC_50_)	Suppression of virus replication	Gong et al. (2013)
Steroids	*Subergorgia suberosa*	H1N1	35.64–50.95 μM (IC_50_)	—	Cheng et al. (2016)
Streptomycetes	*Sarcophyton convolutum*	H1N1 and HCV	—	Suppression of virus replication	El-Gendy et al. (2022)
Lobohedleolide	*Lobophytum crassum*	HCV	10±0.56–22±0.75 μM (EC_50_)	Inhibition of HCV-induced cyclooxygenase-2 (COX-2) expression	Lin et al. (2018)
Tetraphenylbenzoquinone	*Sinularia capillosa*	HCMV	—	—	Cheng et al. (2010a)

^a^ Y refers to the medication

### 4.5 Antibacterial

As shown in Table 5, according to the literature, the antimicrobial activity of coral is mainly exhibited in terms of activity against bacteria (Gram-negative and Gram-positive bacteria, etc.) and fungi. Its antibacterial activity is mainly attributed to terpene compounds extracted from coral, particularly sesquiterpenes and diterpenes, followed by steroidal active substances. In 1997, Badria’s team demonstrated the antibacterial activity of sarcophytolide extracted from soft corals using reagents such as dimethyl sulfoxide and showed that the compound had broad activity against *Staphylococcus aureus*, *Pseudomonas aeruginosa*, *Candida albicans*, and *oenococcus oeni*. Mohamed N. Gomaa not only tested the antibacterial activity of the soft coral of the *Sarcophyton* genus but also compared the differences in the antibacterial activity of different extracts. The results showed that the hexane extract had a strong antibacterial effect. The antibacterial activity of nerve sphingolipids and sterols extracted from *A. dichotoma* ^※^ was also demonstrated using the disc diffusion technique (Al-Lihaibi et al., 2010). The diterpenoids isolated from *Lemnalia* sp. also showed antibacterial activity with MICs of 4–64 μg/mL for *Bacillus subtilis* and *Staphylococcus aureus* (Yan et al., 2021). The antibacterial mechanism has not been specifically reported.

**TABLE 5:** Classification statistics for antibacterial effects of active substances extracted from the coral.

Active ingredient	Source	Strain	Concentration^a^	Reference
Sesquiterpenoids	*Anthogorgia* sp.	*Staphylococcus aureus*	100 μg/mL (Y)	JI and Liu (2018)
Sesquiterpenoids	*Muriceides collaris* ^※^	*Vibrio anguillarum*, *Vibrio harveyi*, and *Vibrio alginolyticus*	0.1, 1, 10, and 100 μg/mL (Y)	Shi (2009)
Sesquiterpenoids	*Litophyton arboreum*	*Bacillus cereus*	1.8 μmol (Y)	Abou El-Kassem et al. (2018)
Sesquiterpenoids	*Paralemnalia thyrsoide*	*Staphylococcus aureus*, *Escherichia coli*, *Candida albicans*, and *Aspergillus niger*	0.221–2.248 µmol (MIC)	Elshamy et al. (2021)
Sesquiterpenoids	*Lemnalia* sp.	*Bacillus subtilis*	4–8 μg/mL (MIC)	Liu et al. (2022)
Sesquiterpenoids	*Xenia* sp.	*Lagenidium thermophilum*	25 μg/mL (MIC)	Phan et al. (2019)
Diterpene	*Junceella juncea* ^※^	Fungi: *Aspergillus niger*, *Candida albicans, and Penicillium notatum*	200 μg/mL (Y)	Murthy et al. (2011)
Diterpene	*Lobophytum pauciflorum*	*Staphylococcus aureus* and *Streptococcus pneumoniae*	20 µg/mL (Y)	Yan et al. (2010)
Diterpene	*Dichotella gemmacea* ^※^	*Staphylococcus albus* and *Staphylococcus aureus*	10–20 μmol/mL (MIC)	Sun (2012)
Diterpene	*Lobophytum* sp.	*Staphylococcus aureus* and *Streptococcus pneumoniae*	—	Zhao et al. (2013a)
Diterpene	*Lemnalia* sp.	*Bacillus subtilis and Staphylococcus aureus*	4–64 μg/mL (MIC)	Yan et al. (2021)
Diterpene	*Dichotella gemmacea* ^※^	Gram-positive bacterium *Bacillus megaterium and* Gram-negative bacterium *Escherichia coli*	0.05 mg (Y)	Li et al. (2016)
Diterpene	—	*Trypanosoma brucei* and *Leishmania donovani*	≤1 μM and <0.2 μM (IC_50_)	Thao et al. (2015)
Diterpene	*Nephthea* sp*.*	*Staphylococcus aureus* and *Escherichia coli*	2.4, 3.0, and 6.0 µg/mL (MIC/MBC)	Ishii et al. (2016)
Terpenoids	*Sarcophyton trocheliophorum*	Gram-positive and Gram-negative bacteria	0.5, 1, 2.5, and 5 mg/mL (Y)	Gomaa et al. (2016)
Steroids	*Sarcophyton* sp.	*Escherichia coli*, *Bacillus megaterium*, *Microbotryum violaceum*, and *Septoria tritici*	—	Wang et al. (2013a)
Steroids	*Carijoa* sp.	*Pseudomonas putida*, *Bacillus cereus*, and *Tetragenococcus halophilus*	31 nM (Y)	Zhao (2013)
Steroids	*Sarcophyton* sp.	*Staphylococcus albus*	20 μmol (Y)	Sun et al. (2013)
Steroids	*Carijoa* sp.	Pseudomonas putida	31 nM (Y)	Zhao et al. (2013c)
Diterpene and steroidal saponin	*Dichotella gemmacea* ^※^	*Bacillus megaterium* and *Botrytis cinerea*	—	Jiang (2013)
Polyphenol	*Talaromyces* sp.	*Escherichia coli*, *MRSA*, *Staphylococcus. aureus*, and *Enterococcus faecalis*	0.45–15.6 μg/mL (MIC)	Li et al. (2021b)
Lobophorin	*Lophelia pertusa*	Pathogenic Gram-positive bacteria such as *Staphylococcus aureus*	40–80 μg/mL (MIC)	Braña et al. (2017)
BCE	*Sarcophyton sp.*	Pathogenic Gram-positive bacteria such as *Staphylococcus aureus* and *Staphylococcus epidermidis*	37 and 73 µg/µL (MIC)	Bai (2011)
—	*Xenia* sp.	*Lagenidium thermophilum*	25 μg/mL (MIC)	Phan et al. (2019)
—	*Nephthea* sp.	*Lagenidium thermophilum*	12.5 μg/mL (MIC)	Tani et al. (2019)
—	*Muricella sibogae*	*Vibrio anguillarum*	0.1, 1, 10, and 100 μg/mL (Y)	Li (2010b)
—	*Sinularia polydactyla*	*Gram-positive bacteria: Bacillus subtil*is and *Bacillus megaterium*	3.9–62.5 μg/mL (MIC)	Aboutabl el et al. (2013)

^a^ Y refers to the medication

### 4.6 Antioxidant activity

Altered oxidative status may have peroxidative effects on lipids, proteins, and RNA and regulate cellular responses, signal transduction, and metabolism, thereby impairing their biological functions. At present, few reports on the antioxidant effect of coral can be retrieved, and the antioxidant effect mostly works through free radical scavenging, oxidative free radicals, and lipid peroxidation. In general, common free radicals include ^−^OH, O^2−^, DPPH, and ABTS^−/+^. The coral derivatives sinularin and dihydrosinularin showed general radical scavenging activity against the free radicals 2,2-diphenyl-1-picrylhydrazyl (DPPH), 2,2-azinobis (3-ethyl-benzothiazoline-6-sulfonic acid) (ABTS), and hydroxyl (-OH), as well as the induction of Fe^+3^ reduction and Fe^+2^-chelating ability, all of which enhanced their antioxidant activity. Sinularin exhibited higher antioxidant properties than dihydrosinularin. Further ATP assays showed that the different antioxidant properties contributed to the antiproliferative effect on different cancer cells as well (Wang et al., 2021). The *in vitro* antioxidant results of the active ingredients BCE (alkanes, terpenoids, esters, fatty acids, and aromatic compounds) extracted from black horn coral^※^ indicated that some of them have scavenging effects on DPPH- and OH-. The *in vivo* antioxidant effect not only induces a morphological protective effect on lung tissue but also effectively increases SOD activity *in vivo* and reduces the MDA content, thereby reducing the damage to lung tissue caused by the large amount of oxygen free radicals in tobacco (Bai, 2011).

### 4.7 Antimalarial

Malaria, caused by *Plasmodium vivax*, poses a major health threat to the majority of the world’s population (Thao et al., 2015). Various marine natural products with anti-protozoal activity have been reported in the literature (Watts et al., 2010; Sanchez et al., 2013; Mohyeldin et al., 2017). Thao et al. (2015) identified laevigatol A in Vietnamese soft corals, which showed inhibition of the *Plasmodium falciparum* (Pf) NF54 strain with IC_50_ < 5.0 µM. The antimalarial activity of sesquiterpene extracts of the octocoral coral *Eunicea* sp.^※^ (Plexauridae: *Octocorallia*: Cnidaria) was demonstrated against chloroquine-resistant strains of *Plasmodium falciparum* by inserting fluorochromes into the parasite DNA. The results revealed that compounds showed a significant inhibition of *Plasmodium falciparum* growth (Garzón et al., 2005). Ospina et al. (2005) conducted an experiment and showed that caucanolide A, a diterpene compound extracted from anise coral, exhibited significant *in vitro* antiplasmodial activity against *Plasmodium falciparum* W2 at an IC_50_ of 17 μg/mL, and caucanolide D was equally effective at an IC_50_ of 15 μg/mL. Please refer to Table 6 for details.

**TABLE 6:** Classification statistics for antioxidant effects of active substances extracted from the coral.

Active ingredient	Source	Mechanism	Concentration	Reference
Sesquiterpenoids	*Sinularia sp.*	Oxidative free radical absorption	5.36 units (1 mM of Trolox equivalent) per 0.31 mg/mL (IC_50_)	Zhang et al. (2006a)
Steroids	*—*	Lipid peroxidation (Vit C/Fe^2+^ excited)	7.6, 30.6, and 122.2 µmol/L (IC_50_)	Xu et al. (1997)
pseudopterosin I	*Sinularia suberosa*	Free radical scavenging:·OH, O^2-^, DPPH	0.1006, 0.1001, and 0.021 mg/mL (IC_50_)	Xiang (2016)
pseudopterosin II	*Sinularia suberosa*	Free radical scavenging:·OH, O^2-^, DPPH	0.2509, 0.2519, and 0.053 mg/mL (IC_50_)	Xiang (2016)
Cladiellin A	*Cladiella sp.*	Oxidative free radical absorption	3.151, 4.781, and 5.171 µM (IC_50_)	Zhang et al. (2005b)
Sinularin	*—*	Free radical scavenging: DPPH, ABTS*•*+, and *•*OH	250–400 µM (Y)	Wang et al. (2021)
Dihydrosinularin	*—*	Free radical scavenging: DPPH, ABTS*•*+, and *•*OH	200–400 µM (Y)	Wang et al. (2021)
Lobocompactols A	*Lobophytum compactum*	Oxidative free radical absorption	1.4 and 1.3 µM Trolox equivalents, respectively, at a concentration of 5 µM (IC_50_)	Chau et al. (2011)
Lobocompactols B	*Lobophytum compactum*	Oxidative free radical absorption	1.4 and 1.3 µM Trolox equivalents, respectively, at a concentration of 5 µM (IC_50_)	Chau et al. (2011)
BCE	*—*	Free radical scavenging: DPPH, *•*OH, and Lipid peroxidation	—	Bai (2011)

### 4.8 Immunosuppressive effect

According to incomplete statistics, terpenoid and sterol active substances extracted mainly from the soft coral *Sinularia scabra*, *Sinularia polydactyla*, *Sinularia* sp., *Libertasomyces* sp., and gorgonian^※^ *Verrucella umbraculum* ^※^ have immunosuppressive effects *in vitro*. Sun et al. (2017) reported for the first time the immunomodulatory activity of new polyketide and trans-fused decane ring system-like metabolites by inducing the proliferation of CD3^+^ T cells. Further structure–activity analysis revealed a key role of the Δ7 and terminal OH groups in the regulation of CD3^+^ T-cell proliferation. Yang et al. (2020) revealed that the sterol compound yalongsterol A, 5α,8α-epidioxy-24-methyl-cholesta-6,24 (28)-dien-3β-ol and (22E,24S)-5α,8α-epidioxy-24-methylcholesta-6,22 -dien-3β-ol, exhibited moderate immunosuppressive activity against T and/or B lymphocytes with semi-inhibitory concentration values of 19.30–59.49 µM. Subsequently, Cui et al. (2020) showed that polycyclic furanobutenolide-derived norditerpenoids exhibited strong inhibitory effects on ConA-induced T lymphocyte and/or LPS-induced B lymphocyte proliferation. Diterpenoids of different membrane types isolated from the South China Sea soft coral *S. scabra* have the same biological activity (Yang et al., 2019). A recent report revealed that metabolites containing the 9,10-secosteroid structure extracted from the South China Sea gorgonian *V*. *umbraculum* ^※^ showed immunomodulatory activity by inhibiting the differentiation of CD4^+^ T lymphocytes (Li J. et al., 2021).

### 4.9 Enzymatic activity

As summarized in Table 7, reports on coral enzyme activity are rare, but from the collected literature, it can be seen that some terpene masses isolated from coral have enzyme inhibitory activity. In addition, some steroid, polyketide, and alkaloid active substances may also have enzyme activity. In-depth research has led to the understanding of the significant role of enzymes in the regulation of diseases, not only for the adjuvant treatment of important organs such as the brain, heart, liver, and kidneys but also in the selective treatment of tumors with remarkable results. The diterpenes sinupol and sinulacetate exhibit good inhibitory activity against protein tyrosine phosphatase 1B (PTP1B), which in turn is a potential drug target for the treatment of type II diabetes and obesity (Ye et al., 2018). Cespine diterpenes isolated from the soft coral *Sinularia crassa* in the South China Sea are used as alpha-glucosidase inhibitors for antidiabetic treatment. This provides a different way of thinking for developing new drugs (Wu et al., 2020b).

**TABLE 7:** Classification statistics for enzymatic activity of active substances extracted from the coral.

Active ingredient	Source	Related active substance	Concentration^a^	Effect	Reference
Sesquiterpenoids	*Sinularia* cf. *molesta*	PTP1B	1.24 μmol/L (IC_50_)	Inhibitor	Chu et al. (2018)
Diterpene	*Sarcophyton trocheliophorum*	PTP1B	6.97 μmol/L (IC_50_)	Inhibitor	Liang et al. (2013)
Diterpene	*Sinularia crassa*	α-Glucosidase	10.65±0.16, 30.31±1.22 μmol/L (IC_50_)	Inhibitor	Wu et al. (2020b)
Diterpene	*Sinularia polydactyla*	PTP1B	51.8–72.4 μmol/L (IC_50_)	Inhibitor	Ye et al. (2018)
Diterpene	*Sarcophyton glaucum*	Cytochrome P450 1A	1 µg/mL (Y)	Inhibitor	Hegazy et al. (2012)
Diterpene	*Sarcophyton glaucum*	Glutathione *S*-transferases (GST), quinone reductase (QR), and epoxide hydrolase (mEH)	10 µg/mL (Y)	Inducer	Hegazy et al. (2012)
Diterpene	*Sarcophyton trocheliophorum*	Acetylcholinesterase	40 μmol/L (Y)	Inhibitor	He (2013)
Diterpenoid alkaloids	*Ellisella robusta* ^※^ and *Ellisella curvata* ^※^	Tyrosine kinase c-Met	10 μmol/L (Y)	Inhibitor	Zhang (2012b)
Steroids	*Sinularia dissecta*	COX-2 (cyclooxygenase-2)	7.04 ± 1.03 μmol/L (IC_50_)	Inhibitor	Jin (2005)
Prostaglandin	*Plexaura homomalla*	P38α-kinase, Src-kinase, and topoisomerase IIα	2.5 and 10 μmol/L (Y)	Inhibitor	Hurtado et al. (2020)

^a^ Y refers to the medication.

### 4.10 Effects on the nervous system

The neuroprotective effects of coral are manifested in two ways. On the one hand, they exhibit anticonvulsant and antiepileptic effects. As early as 1984, preliminary pharmacological experimental studies on the soft coral *Lemnalia exilis* showed that its extract had a significant antispasmodic effect on the isolated ileum of guinea pigs (Fang and Zhang, 1984). Eltahawy et al. (2015) measured the anticonvulsant activity of ceramide isolated from the Red Sea soft coral *Sarcophyton auritum* using a pentylenetetrazol (PTZ)-induced seizure model, and the mechanism may be through the modulation of CNS inhibitory activity through GABA and serotonin receptors. Some sterols also exhibited neuroprotective activity against neuron-like SH-SY5Y cells (Tammam et al., 2020). On the contrary, it has a sedative–hypnotic effect (Liao et al., 1992). Finally, the coral derivative excavatolide B can enhance long-term induction by suppressing the delayed rectifier potassium current, which lowers the action potential onset threshold and ultimately enhances situational memory retrieval in mice, resulting in enhanced memory extraction.

The effects of formulated preparations of coral on the nervous system have also been documented. First, Ershiwuwei Shanhu pills can prolong the latency period of epileptic seizures, shorten the duration of epileptic seizures, reduce the level of epileptic seizures, decrease the number of clonic seizures, and suppress epileptic discharges. At a certain dose, its effect was significantly better than that of the positive control drug sodium valproate (Luo, 2012; Luo et al., 2013). Second, Li et al. (2014) explored the protective effects of Ershiwuwei Shanhu pills on senescent hippocampal cells. The drug inhibited D-lactose-induced neuronal degeneration and excessive activation of astrocytes, thereby reducing neuronal and astrocyte damage. Finally, Ershiwuwei Shanhu capsules can increase adenosine levels in secondary spinal cord injury, thereby increasing the ability of nerve cells to repair themselves (Jiao et al., 2013).

### 4.11 Effects on the cardiovascular system

Fang and Zhang (1984) found that soft coral extract has high physiological activity on the cardiovascular system. The extract of soft coral can not only delay the time of arrhythmia in isolated hearts of rats and shorten the duration of arrhythmia but also increase rabbit’s heart coronary flow and slow down the heart rate. Lai (2017) also pointed out that red coral could regulate TXB_2_/6-keto-PGF_1α_ levels, reduce plasma PF_4_/β-TG levels, and lower plasma ET-1 levels in a blood stasis rat model, ultimately reducing vascular injury in rats. 15-Hydroxy-tetracosa-6,9,12,16,18-pentaenoic acid and sesquiterpenes isolated from the soft coral *S numerosa* and *Lemnalia* sp. exhibit anti-tubulinogenic and pro-angiogenic activities, respectively, in a dose-dependent manner (Yao et al., 2007; Yamashita et al., 2009; Wang et al., 2020).

### 4.12 Other effects

Other effects of corals include antihypertensive, hypolipidemic, and antiulcer activities. The diterpene glucoside isolated from the soft coral *Cespitularia turgida* in the South China Sea has a significant acute antihypertensive effect, and it has an obvious quantity–effect relationship; its antihypertensive effect has no rapid tolerance phenomenon, and at the same time, it has little effect on the heart rate when used as antihypertensives. The formulated preparation of coral, Shanhu Qishiwei pill, may reduce blood lipid levels in HLP model rats by inhibiting the LKB1/AMPK signaling pathway (Chun et al., 2022). Elshamy et al. (2017) demonstrated the antiulcer activity in a rat ulcer model induced by ethanol and acetic acid.

## 5 The toxicity of coral

Many corals, such as animal corals, also known as soft corals, are very popular in aquariums (home or public) because of their appreciation value and low maintenance costs. The soft corals of genera *Palythoa*, *Protopalythoa*, *Zoanthus*, and *Parazoanthus* in the Zoanthidae family contain a highly toxic and potentially lethal compound, palytoxin (Hoffmann et al., 2008). Therefore, the toxic compound of coral is mainly palytoxin. Ciminiello et al. (2011) extracted palytoxin and 42-hydroxy palytoxin at levels up to 25–450 ng per kg of Zoanthid. Palytoxin is a potent vasoconstrictor, and its neurotoxicity and cardiotoxicity are primarily due to dysregulation of the transmembrane pump Na/K-ATP enzyme, which can lead to serious human disease, causing gastrointestinal symptoms, myalgia, muscle spasms, respiratory and cardiac problems, and even death (Wieringa et al., 2014). The toxin is heat-resistant, and conventional boiling inactivation operations are not effective against it. Reports of human exposure to palytoxin consumption have described significant morbidity and mortality (Sud et al., 2013).

Palytoxin exposure and the production of toxic compounds through corals are primarily associated with toxin poisoning from inhalation of toxin-dissolved water aerosols during cleaning, scrubbing, or eradication of corals in home/public aquariums. Thus, aquarium store staff and home aquarium hobbyists face a consequent elevated risk of exposure. The data we collected showed that people aged less than 80 years and children exposed to palytoxin nebulized from coral had immediate symptoms such as cough, dyspnea, chest pain, myalgia, tachycardia, and gastrointestinal symptoms, and in severe cases, acute reactions such as burning or stinging and erythema also occur. Coral injuries may also have complications such as foreign body reactions, bacterial infections, or local eczema reactions (Na et al., 2008). Examples of poisoning due to prolonged and unprotected exposure to corals have also been reported (Smith et al., 2003; Hoffmann et al., 2008). A patient who placed his right hand on a Zoanthid colony while cleaning a seawater aquarium at home developed myalgia, symptoms of general weakness in limbs, and, subsequently, signs of poisoning such as speech impairment, dull eyes, and fainting. The degree of poisoning is closely related to the contact time, contact distance, and contact method. Subsequently, corneal toxicity due to exposure to Zoanthid corals has been documented. Seven patients presented with corneal manifestations ranging from superficial punctate epithelial lesions to bilateral corneal melting and subsequent perforation, with some patients presenting with progressive corneal melting even requiring therapeutic penetrating corneal transplantation. Fortunately, more than half of these case reports show that short-term minor injuries are reversible with medication or emergency measures, with only a few disabilities or a significant reduction in quality of life due to sequelae (Chang et al., 2020).

In 2014, water extracts from water corals were first reported to contain a lethal nonpeptide neurotoxin (García-Arredondo et al., 2015). The investigators administered 5.3 µg protein/g body weight of the extract to mice intravenously, which caused violent convulsions and death in the range of 1 min and histopathological damage to the kidneys and lungs at doses below the LD_50_ (LD_50_ = 4.62 µg protein/g body weight). After incubation under heat denaturing conditions, this histopathological damage was completely eliminated. However, the denatured extracts maintained their lethal effect. Second, in the process of researching the anti-neurotoxic active ingredients of the side flat soft willow coral, it was found that water-insoluble parts of alkali extracts of *S. suberosa* can make the animal produce a whole body soft, heavy limb tremor, turn positive reflex disappear, and cause other reactions (Liao et al., 1992).

Coral is often used as medicine in combination. Ershiwuwei Shanhu pills and others are classic Tibetan remedies consisting coral preparations. In the acute toxicity test of Ershiwuwei Shanhu pills, there were no obvious acute toxic reactions, but in the subacute toxicity test, toxic damage to liver, kidney, and lung pathological sections was observed (Liu F. L. et al., 2016). Long-term doses of Ershiwuwei Shanhu pills lead to accumulation of copper, mercury, and lead in the internal organs of the rats, with few rats developing symptoms of the vegetative nervous system, such as increased salivary gland secretion (LI, 2011). It can cause toxic reactions, manifested in immune function, and liver, kidney, and lung tissues are affected and damaged to varying degrees. The main toxic target organs are the liver, kidney, and lung, and damage due to toxicity occurs in a dose-dependent manner (Liu F. L. et al., 2016). However, given the complexity of its compounds, specific toxic substances remain to be investigated.

## 6 Clinical application

### 6.1 Individual application of coral

Coral’s good stability, ease of use, and low cost contribute to its use as main material in the treatment of orthopedic diseases. In addition, coral contains 11 kinds of trace elements, namely, Zn0.05, Cu0.6, Pb0.0025, Ni0.004, Ti0.005, Mn0.004, Fe0.7, Al0.35, Mg3, Si > 1.0, and Sr0.1, and most of these trace elements are indispensable to the human body (Wang et al., 2002b). Xiao et al. (2005) systematically reported on black horn coral for the treatment of bone injury diseases. After taking the medicine for 5–7 days in mild cases and 1–2 months in severe cases, patients’ clinical symptoms were basically relieved, and X-ray films showed that the bone changes were basically corrected or in a stable state. In the clinical method of immediate implant placement, artificial coral bone powder particles were placed in the bone defect area near the crest of the alveolar fossa, where significant osteogenesis was observed after 6 months. The gingival texture and color were better than before the restoration (Zhou, 2014).

Coral clinical applications are detailed in Table 8. It is often processed into powder for punching or used directly to treat bone injury diseases. It is also very effective in the treatment of cerebral vascular sclerosis and coronary artery sclerosis (Yuan, 1991). In 1990, the School of Medicine of Kyoto University in Japan extracted a substance from the coral of the cockle and used one 100th of a gram of it to mix into 1,000 mL of compound saline for injection or infusion. In difficult cases, it is also often used in combination with restorative dental tablets. However, the mechanism of action of coral is still unknown to us. In the available literature, it has been reported that it may be related to the absorption of coral by osteoclast-associated proteins (Lin Y. Y. et al., 2013) and bone marrow granulation tissue and blood vessels (Guillemin et al., 1989). However, it is also only a vague term, and a clearer and more explicit mechanism has to be studied.

**TABLE 8:** Classification statistics for individual application of coral.

Disease	Pharmaceutical preparation	Experimental subject	Research design	Groups and number of people		Therapeutic method		Course of treatment	Curative effect	Reference
				Treatment group	Control group	Treatment group	Control group			
Bone defects and non-union	Deer horn coral skeleton	35 cases: 32 males and 2 females	Randomized controlled trials	35 cases: 32 males and 2 females	—	Coral bone particles are disinfected under high pressure and placed at the desired bone graft site in the human body	—	—	—	Wang et al. (2002b)
Avascular necrosis of the femoral head, bone hyperplasia, and spinal and lumbar lesions	Black horned coral skeleton	23 cases: 14 males and 9 females	Randomized controlled trials	23 cases: 14 males and 9 females	—	Crush the black horn coral and add softener to form a powder and take it in the herbal soup	—	1 dose per day for 20–30 days	One case died of cerebrovascular accidental death, and the other 22 cases were examined 1∼2 months after taking the medicine. The clinical symptoms basically disappeared or relieved; particularly, the osteoporosis was basically corrected or stabilized	Xiao et al. (2005)
Patients with residual roots of anterior teeth and premolars	Coral bone powder	34 cases: 19 males and 15 females	Randomized controlled trials	34 cases: 19 males and 15 females	—	Artificial coral bone powder particles are implanted and undergo secondary repair surgery through porcelain crowns 6 months later	—	1 year	34 patients had significant bone formation in their alveolar ridges before the second-stage surgery. After the second-stage repair, they recovered normally, and the texture and color of the gums were better than before the repair	Zhou (2014)
Extraction of molars	Coral bone powder	45 cases: 23 males and 22 females	Randomized controlled trials	20 cases: 10 males and 10 females	25 cases: 13 males and 12 females	Fill the extraction socket with coral bone powder, and perform restoration operations such as filling the amount flush with the top of the adjacent alveolar ridge	Conventional biting gauze roll for approximately 30 minutes	6 months	The gingiva on the buccal and lingual sides of the experimental group was smooth and continuous, forming a plateau shape, and the alveolar bone was plump; the height and width of the alveolar bone in the control group were significantly reduced, and the buccal lingual side of the occlusal surface was significantly sunken, resulting in a narrow and elongated alveolar bone. After 6 months of tooth extraction, the degree of alveolar ridge atrophy in the experimental group was lower than that in the control group	Liu and Wang, (2014s)
Nasal deformity	Black horned coral skeleton	20 cases: 12 males and 8 females	Randomized controlled trials	20 cases: 12 males and 8 females	—	External nose shaping technology and implanting appropriately carved coral blocks into the nasal cone	—	—	18 patients recovered smoothly, 1 had an unexpected fracture, and 1 had an infection	Dagli et al. (1997)
Delayed sternal closure	Coral hydroxyapatite	1 male	Randomized controlled trials	1 case: 1 male	—	—	—	—	—	Zacharias et al. (2004)
Cranial injury or postoperative repair	Coral fragments	72 cases	Randomized controlled trials	72 cases	—	—	—	17 months	50% of cases have coral skeletons almost completely absorbed; another 50% of cases have partial absorption. The absorption of coral structures in larger implants does not exceed 40% of their volume, and no infectious complications have been found	Roux et al. (1988)
Craniofacial bone contour defect	Coral fragments	36 cases: 13.39% males and 22.61% females	Randomized controlled trials	—	—	—	—	12–36 months	Except for five clinically significant material absorption sites (incomplete absorption), the enhancement effect of other patients is very stable	Marchac and Sandor (1994)
Cervical adjacent segment degenerative disease	—	52 cases: 37 males and 15 females	Randomized controlled trials	52 cases: 37 males and 15 females	—	Eleven patients underwent anterior cervical discectomy and fusion (ACDF), 24 patients underwent anterior cervical discectomy and fusion (ACDF), and four patients underwent cervical disc replacement (CDA). The median time interval between the first and second surgeries was 74 months	Thirteen patients underwent their first SLAC surgery. The median time interval between the first and second surgeries was 33 months (21–59 months)	—	—	He et al. (2022)
Cerebrovascular sclerosis, coronary arteriosclerosis, and heart disease	Cockscomb coral extract	—	Randomized controlled trials	—	—	One part per million of this substance is refined and mixed with 1,000 ml of compounded saline for injection or infusion to patients with significant therapeutic effects	—	—	—	Yuan (1991)

### 6.2 Clinical application of preparations that contain coral

In clinical practice, the compound prescription of coral is mainly composed of Ershiwuwei Shanhu pills, Ershiwuwei Shanhu capsules, and Shanhu Qishiwei pills. Ershiwuwei Shanhu pills are a traditional, famous prescription and proven recipe for Tibetan medicine to treat albichoriasis and epilepsy. It uses coral as the monarch drug, together with pearl, *Terminalia chebula* and so on. It restores nerve function and relieves pain. It is mainly used to treat albichoriasis, unconsciousness, body numbness, dizziness, brain pain, irregular blood pressure, headache, epilepsy, and various types of neuropathic pain. Based on the collected literature, Ershiwuwei Shanhu pills has satisfactory clinical efficacy in the treatment of neurological diseases (epilepsy, primary headache, etc.), cardiovascular diseases (cerebral infarction, hypertension, etc.), and orthopedic system (neurogenic cervical spondylosis, lumbar myofasciitis, etc.). In acute and severe cases, the combination of drugs is often used clinically to promote a synergistic effect and relief (Table 9).

**TABLE 9:** Classification statistics for clinical application of preparations that contain coral.

Disease	Pharmaceutical preparation	Experimental subject	Research design	Groups and number of people		Therapeutic method		Course of treatment	Curative effect	Reference
				Treatment group	Control group	Treatment group	Control group			
Cervical spondylosis of vertebral artery type	Ershiwuwei Shanhu capsules combined with Western medicine	84 cases: 54 males and 30 females	Randomized controlled trials	42 cases: 28 males and 14 females	42 cases: 26 males and 16 females	On the basis of the control group, add 2 capsules of Ershiwuwei Shanhu that contains coral each time, with a specification of 0.5 g per capsule, once a day	Routine treatment of Western medicine: oral flunarizine hydrochloride capsules every 10 mg, once a day, before sleep, and chiropractic treatment	30 days	Observation group: 22 cases were cured, 10 cases were significantly improved, six cases were effective, and four cases were ineffective, with a total effective rate of 90.48%. Control group: 14 cases were cured, six cases were significantly improved, nine cases were effective, and 13 cases were ineffective, with a total effective rate of 69.05%. The pain score decreased after treatment, and the decrease in the observation group was better than that in the control group	Ren et al. (2015)
Cervical spondylosis of vertebral artery type	Ershiwuwei Shanhu capsules	90 cases	Randomized controlled trials	45 cases	45 cases	On the basis of the control group, orally take 2 capsules of Ershiwuwei Shanhu that contains coral per day for 20 days as a course of treatment	Chiropractic treatment [spinal neurobiomechanical reduction method (found by Luo Xiaoyang)], once every 3–4 days, 5 times as a course of treatment	—	After treatment, the two subgroups showed improvements in relieving neck and arm pain, neck tenderness, cervical mobility, and upper limb numbness compared to before treatment, with the combined treatment group showing more significant improvements	Li et al. (2013b)
Cervical spondylosis	Ershiwuwei Shanhu pills	65 cases: 23 males and 42 females	Randomized controlled trials	65 cases: 23 males and 42 females	—	Twenty-five flavor coral pills, taken orally, at the same time, according to the condition and combined with acupuncture and moxibustion treatment, a course of 10 days, generally 2–3 courses	—	20–30 days	444 cases recovered without any clinical symptoms, 15 cases were effective, and six cases were ineffective. The total effective rate is above 90%	Zhang and Zhang (2011)
Lumbar fasciitis	Ershiwuwei Shanhu pills	150 cases: 98 males and 52 females	Randomized controlled trials	150 cases: 98 males and 52 females	—	Take Ershiwuwei Shanhu pills, 4 pills per dose, 1 dose per day, with warm water-soaked powder and medication residue on an empty stomach.	—	21 days	Cured 30 people by 20%; improved 108 people by 72%; 11 people had no significant changes, accounting for 7.3%; one person has not recovered, accounting for 0.6%, indicating aggravation	Li (2006)
Epilepsy	Ershiwuwei Shanhu pills	136 cases: 62 males and 74 females	Randomized controlled trials	68 cases: 32 males and 36 females	68 cases: 30 males and 38 females	Oral administration of Ershiwuwei Shanhu pills, 1 g each time, once a day, with warm water	Taking anti-epileptic drugs, including 20 cases treated with single drug, 38 cases treated with dual drug, and 10 cases treated with combination of three drugs	2 months	The total effective rate of the treatment group who only took Ershiwuwei Shanhu pills was significantly higher than that of the control group	Wang et al. (2014a)
Epilepsy	Ershiwuwei Shanhu pills	112 cases: 65 males and 47 females	Randomized controlled trials	56 cases: 34 males and 22 females	56 cases: 31 males and 25 females	Oral Tibetan medicine Ershiwuwei Shanhu pills for treatment, 1 g/time, 1 time/day, taken with warm water	Oral administration of sodium valproate tablets, 0.2–0.4 g/time, 3 times/day, or additional administration of topiramate tablets (25–200 mg/time, 2 times/day) or phenytoin sodium tablets (50–100 mg/time, 2–3 times/day)	2 months	The total effective rate of the Ershiwuwei Shanhu pills group was 91.07%, while the total effective rate of the control group was 67.86%	Wang et al. (2014b)
Epileptic tonic–clonic seizures	Ershiwuwei Shanhu pills	102 cases: 62 males and 40 females	Randomized controlled trials	51 cases: 32 males and 19 females	51 cases: 30 males and 21 females	Oral administration of Ershiwuwei Shanhu pills, 1 g each time, once a day, with warm water	Sodium valproate tablets, 0.2–0.4 g/time, 3 times/day, or topiramate tablets (25–200 mg/time, 2 times/day) or phenytoin tablets (50–100 mg/time, 2–3 times/day)	2 months	The total effective rate of the treatment group was 88.23%, while the total effective rate of the control group was 68.62%. Compared with before treatment, the epilepsy symptom scores of both groups were significantly reduced after treatment. Compared with the control group, the symptom scores of the treatment group were significantly reduced	Wang, Z.S. et al. (2013)
Epilepsy	Ershiwuwei Shanhu pills combined with carbamazepine	82 cases	Randomized controlled trials	41 cases	41 cases	On the basis of the control group, oral administration of Ershiwuwei Shanhu pills, 1 pill/time, 1 dose/day	Take orally carbamazepine tablet, the initial dose is 0.2 g/time, twice a day. After one week of continuous treatment, adjust the dose, increase by 0.1 g per week, to 0.4 g per time, twice a day	2 months	After treatment, the total effective rates of the control group and the treatment group were 80.95% and 95.24%, respectively. The HAD scores of both groups were significantly reduced, while MoCA was significantly increased. The number of epileptic seizures in both groups of patients was significantly lower than before treatment, and the serum levels of IL-2 and TNF-α in both groups of patients were significantly lower; the reduction of the above indicators in the treatment group was better than that in the control group	Huang and Zhao (2017)
Epilepsy	Ershiwuwei Shanhu pills combined with levofloxacin tablets	60 cases	Randomized controlled trials	30 cases	30 cases	On the basis of treatment in the control group, oral administration of Ershiwuwei Shanhu pills, 1 g/time, 1 time/day	Oral administration of levetiracetam tablets after meals, starting at a dose of 500 mg/time, twice a day, and adding to 1,000 mg/time, twice a day after one week	3 months	The total effective rates of the control group and the treatment group were 73.33% and 93.33%, respectively, and the levels of inflammatory factors in the treatment group were significantly lower than those in the control group. After treatment, the frequency of seizures in both groups was significantly reduced, and the frequency of seizures in the treatment group was significantly lower than that in the control group	Yuan et al. (2018)
Epilepsy	Combined use of Ershiwuwei Shanhu pills	176 cases: males:females=2:1	Randomized controlled trials	—	—	The addition group of Wuwei Coral pills was composed of carbamazepine, valproic acid, and Xilishu	Valproic acid added with Shunqi Anshen Wan	1 year	Three compatibility schemes of Ershiwuwei Shanhu pills (three groups of Ershiwuwei Shanhu pills addition group) have a significant effect on reducing the frequency of seizures, alleviating the degree of epileptic discharge, and improving the degree of headache and cognitive impairment after seizures in symptomatic epilepsy. Among them, the combination of Ershiwuwei Shanhu pills and sodium valproate group and the combination of Xilishu group both have the effect of improving the type of epileptic seizures. The combination of Xilishu group can also significantly shorten the duration of epileptic seizures	Luo (2012)
Primary headache	Ershiwuwei Shanhu capsules combined with cowpox vaccine	67 cases: 26 males and 41 females	Randomized controlled trials	30 cases: 12 males and 18 females	37 cases: 14 males and 23 females	Ershiwuwei Shanhu capsules, 4 capsules per day, intravenously administered with a dose of 3 ml of rabbit skin extract induced by cowpox vaccine, added to 5% glucose injection (250 ml) once a day	Conventional Western medicine treatment, oral fluranolol cinnarizine 1 capsule per night, intravenous drip of Venoruton 250 ml, once a day	2 weeks	Observation group: 18 cases showed significant effect, 10 cases were effective, and two cases were ineffective, with a total effective rate of 93.3%; Control group: 16 cases showed significant effect, 14 cases were effective, and seven cases were ineffective, with a total effective rate of 81.1%; The headache relief rate in the study group was higher than that in the control group	Bian et al. (2016)
Migraine	Ershiwuwei Shanhu pills	50 cases: 13 males and 37 females	Randomized controlled trials	30 cases: 8 males and 22 females	20 cases: 5 males and 15 females	Ershiwuwei Shanhu pills, 4 pills per time, once a day	Sibeline 10 mg, once a day; 10 mg of oryzanol, three times a day; Qiye Shen'an tablets 100 mg, three times a day	4 weeks	The total effective rate of the treatment group was 93.33%. The total effective rate of the control group was 75%	Huang (2008)
Migraine	Ershiwuwei Shanhu pills	40 cases: 12 males and 28 females	Randomized controlled trials	40 cases: 12 males and 28 females	—	Ershiwuwei Shanhu pills, 4 at a time, once a day	—	1 month	Twelve cases were cured, accounting for 30.0%; 17 cases showed significant effect, accounting for 42.5%; eight cases were effective, accounting for 20.0%; three cases were ineffective, accounting for 7.5% of the total. Total effective rate was 92.5%	Yang (2010)
Migraine	Ershiwuwei Shanhu pills	480 cases: 211 males, 269 females	Randomized controlled trials	235 cases: 111 males, 124 females	245 cases: 100 males, 145 females	Ershiwuwei Shanhu pills 3 tablets/1, 2 times/d, swallowed in installments	Ershiwuwei Shanhu pills 4 tablets/1, 1 time/day, taken by soaking in hot water	4 weeks	The cure rate in the conventional dose group was 115/245 cases, while the cure rate in the high-dose group was 148/235 cases	Zhou (2009)
Migraine	Ershiwuwei Shanhu pills in combination with flunarizine	112 cases: 49 males and 63 females	Randomized controlled trials	56 cases: 26 males and 30 females	56 cases: 23 males and 33 females	Twenty-five flavor coral pills, 4 pills each time (0.25 g each), once a day, fluranine cinnarizine capsules 5 mg, taken daily before sleep	Flunarizine 5 mg, taken daily before bed	4 weeks	After treatment, the peak systolic period in the treatment group improved significantly compared to before treatment	Zhao (2011)
Migraine	Ershiwuwei Shanhu pills combined with sibeline	158 cases: 56 males and 102 females	Randomized controlled trials	84 cases: 27 males and 57 females	74 cases: 29 males and 45 females	Ershiwuwei Shanhu pills, taken orally in warm water every morning, 4 capsules per dose; take 1 sibeline capsule before bedtime every night	On the basis of conventional medication treatment, sibeline is administered orally, taking 1 capsule before bedtime every night	4 weeks	The observation group significantly alleviated the level of anxiety or depression in patients, with better results than the control group	Chen et al. (2014a)
Migraine	Ershiwuwei Shanhu pills combined with acupuncture and moxibustion	110 cases: 37 males and 73 females	Randomized controlled trials	55 cases: 20 males and 35 females	55 cases: 17 males and 38 females	Acupuncture and moxibustion treatment and taking Ershiwuwei Shanhu pills, 1 g/time, once a day.	Acupoint selection: Select the acupoints on the patient’s diseased side, such as Baihui, Shenting, Benshen and Lugu, as well as other acupoints such as Waiguan, Fengchi, and Jiaosun Qiuxu. Acupuncture at different acupoints for different diseases. Patients with liver disease may experience symptoms by needling the Taichong and Xingjian acupoints. For patients with blood deficiency, the Xuehai and Sanyinjiao acupoints should be added. For patients with kidney deficiency, the Guanyuan and Taixi acupoints should be added. For patients with blood stasis, the Quchi and Hegu acupoints should be added. After obtaining qi, use the technique of calming and tonifying and reducing the symptoms, and leave the needle for 30 minutes, once per day	5 weeks	The total effective rate of clinical efficacy was 94% in the observation group and 80% in the acupuncture and moxibustion group, and the observation group is superior to the acupuncture and moxibustion group	Gu (2014)
Stubborn headache	Ershiwuwei Shanhu pills	128 cases: 78 males and 50 females	Randomized controlled trials	64 cases: 40 males and 24 females	64 cases: 38 males and 26 females	Ershiwuwei Shanhu pills, 1 g each time, once a day, taken with warm water	Oral Zhengtian pills, 1 bag (6 g) each time, 3 times a day, discontinue other medications and painkillers 1 week before and during treatment	8 weeks	The frequency, intensity and duration of pain in the treatment group were significantly lower than those in the control group; the total effective rate of the treatment group was 93.75%. The total effective rate of the control group was 81.25%	Wang et al. (2014c)
Stubborn headache	Ershiwuwei Shanhu pills	80 cases: 47 males and 33 females	Randomized controlled trials	40 cases: 26 males and 14 females	40 cases: 21 males and 19 females	Ershiwuwei Shanhu pills, taken in boiling water, 1 g once, twice a day	Take Zhengtian pills orally, once in the morning, once in the afternoon, once in the evening, and take one bag each time. Take amitriptyline hydrochloride tablets in combination, once in the morning and once in the evening, taking 2 tablets each time	1 month	After treatment, the pain intensity and duration of the control group patients were higher than those of the observation group, with a total effective rate of 72.5% in the control group and 92.5% in the observation group	Wang (2016)
Stubborn headache	Ershiwuwei Shanhu pills combined with acupuncture and moxibustion	8 cases: 2 males and 6 females	Randomized controlled trials	8 cases: 2 males and 6 females	—	0.6 g per pill, once a day, one pill per time. Take one pill at night and soak it overnight with a little saffron and bear bile, then take it at dawn the next day. Acupuncture should be done once a day for the initial treatment, which can be combined with moxibustion. Change to acupuncture and moxibustion every other day after pain relief	—	—	—	Bai and You (2000)
Tension headache	Ershiwuwei Shanhu pills	120 cases: 43 males and 67 females	Randomized controlled trials	55 cases: 22 males and 33 females	55 cases: 21 males and 34 females	4 pills (1 g) each time, once a day, ground and taken with warm water	Flunarizine hydrochloride capsules, 5 mg each time, twice a day	4 weeks	The total efficacy of the treatment group was 54.55%, while that of the control group was 29.09%	Wang, Z.S. et al. (2013)
Tension headache	Ershiwuwei Shanhu pills	70 cases	Randomized controlled trials	35 cases	35 cases	Ershiwuwei Shanhu pills 1 g, oral once a day	Amitriptyline tablets, 25 mg, taken orally 3 times a day	28 days	The total effective rate of the Ershiwuwei Shanhu pill treatment group was 82.86%, while the total effective rate of the amitriptyline control group was 80.00%; the total effective rate of traditional Chinese medicine syndrome in the treatment group was 88.57%, while that in the control group was 82.86%; the effect of the treatment group is better than that of the control group	Dai (2010)
Tension headache	Delixin combined with Ershiwuwei Shanhu pills	160 cases: 58 males and 102 females	Randomized controlled trials	80 cases: 31 males and 49 females	80 cases: 27 males and 53 females	Takes 1 tablet of dailixin orally in the morning and 1 tablet orally in the middle of the day, and 4 capsules of Jinzhu Yalong Ershiwuwei Shanhu pills are taken orally once in the morning	Take 1 tablet of Xi bi ling every night before going to bed, and add symptomatic medications (such as general painkillers, nourishing blood and clearing brain granules, and Tongtian oral liquid)	2 weeks	Among the 80 cases in the treatment group, 80 cases were effective with a total effective rate of 100%, while in the control group, 22 cases were effective with a total effective rate of 85%	Li (2007)
Chronic tension-type headache	Ershiwuwei Shanhu pills combined with low-dose trazodone hydrochloride tablets	120 cases: 26 males and 94 females	Randomized controlled trials	60 cases: 11 males and 49 females	60 cases: 15 males and 45 females	Ershiwuwei Shanhu pills 1 g, once a day (taken in hot water), trazodone hydrochloride tablets 25 mg, once a night	Amitriptyline hydrochloride tablets 25 mg, once per night, gradually increased according to patient tolerance (≤75 mg per day)	3 months	The total effective rate of the treatment group was 81.67%, which was better than the control group's total effective rate of 73.33%; VAS: The observation group showed a better decrease in scores than the control group; HAMD and HAMA: After treatment, the scores of both groups decreased significantly, and the observation group was better than the control group	Zhou et al. (2019)
Frequent episodes of tension-type headache	Ershiwuwei Shanhu pills combined with low-dose amitriptyline	240 cases: 92 males and 148 females	Randomized controlled trials	120 cases: 47 males and 73 females	120 cases, 45 males, 75 females	Take 4 capsules (1.0 g) of Ershiwuwei Shanhu pills orally and soak them in water once a day; amitriptyline tablets 12.5 mg. Twice daily	Amitriptyline tablets 25 mg, twice daily	12 weeks	The total effective rate of the treatment group was 93.33%. The total effective rate of the control group was 73.33%. The therapeutic effect of the treatment group was better than that of the control group	Li (2012b)
Angioneurotic headache	Combination of Ershiwuwei Shanhu pills and nursing intervention	60 cases: 37 males and 23 females	Randomized controlled trials	30 cases: 19 males and 11 females	30 cases: 18 males and 12 females	Take 2 Tibetan medicine Ershiwuwei Shanhu pills once a day, orally before meals; nursing interventions	Zhennaoling treatment: 4 capsules of Zhennaoling each time, three times a day, in the morning, mid-day, and evening, taken orally	30 days	Among the study group of patients, there were 12 controlled cases, seven significantly effective cases, eight effective cases, and three ineffective cases, with a total effective rate of 90.00%; in the control group, eight patients were under control, six were significantly effective, five were effective, and 11 were ineffective, with a total effective rate of 63.33%	He (2017)
Angioneurotic headache	Ershiwuwei Shanhu pills combined with nursing intervention	80 cases: 43 males and 37 females	Randomized controlled trials	40 cases: 22 males and 18 females	40 cases: 21 males and 19 females	Ershiwuwei Shanhu pills, combined with nursing interventions for treatment, dosage is 2 capsules, once a day, administered orally before meals	The dosage of aspirin enteric-coated tablets is 30 mg, 3 times a day, administered orally; the dosage of nimodipine is 30 mg, 3 times a day, administered orally	30 days	Observation group: Among the 40 cases, 28 were significantly effective, 11 were effective, and one was ineffective, with a total effective rate of 97.5%. Control group: Among the 40 cases, 21 were significantly effective, 10 were effective, and 9 were ineffective, with a total effective rate of 77.5%	Li (2021)
Angioneurotic headache	Ershiwuwei Shanhu pills	63 cases	Randomized controlled trials	33 cases	30 cases	Ershiwuwei Shanhu capsules, 2 capsules (0.5 g/capsule), once a day	Zhennaoling, 4 capsules (0.3 g/capsule), 3 times daily	30 days	Thirty cases in the treatment group were effective, with a total effective rate of 90.9%, and 22 cases in the control group were effective, with a total effective rate of 73.3%	Renwang and Renqing (2010)
Poststroke headache	Ershiwuwei Shanhu capsules	70 cases: 47 males and 23 females	Randomized controlled trials	35 cases: 25 males and 10 females	35 cases: 22 males and 13 females	Tibetan medicine Ershiwuwei Shanhu capsules, 2 capsules/time	Routine medical symptomatic treatment should be carried out with antiplatelet aggregation, analgesia, nutritional nerve, and softening vascular drugs	8 weeks	The frequency of headaches in both groups was lower than before treatment, and the duration of pain was shorter than before treatment. The frequency of headaches in the Tibetan medicine group was lower than that in the reference group, and the duration of pain was shorter than that in the reference group	Guan (2020)
Poststroke headache	Ershiwuwei Shanhu capsules	64 cases: 33 males and 31 females	Randomized controlled trials	32 cases: 18 males and 14 females	32 cases: 15 males and 17 females	Twenty-five coral capsules, 0.5 g pellets at 1 time/day and 2 pellets/time, were given orally on the basis of routine internal medicine treatment	Routine medical treatment is given, which includes nourishment of nerves, invigorating blood circulation and eliminating stasis, anti-platelet aggregation, anti-arteriosclerosis effect, and pain relief	4 weeks	The effective rate of the treatment group was 93.75%, while the control group was 56.25%	Shi and Zheng (2018)
Headache	Ershiwuwei Shanhu capsules combined with Danzhen headache capsules	76 cases: 35 males and 41 females	Randomized controlled trials	38 cases: 17 males and 21 females	38 cases: 18 males and 20 females	Combination therapy of Tibetan medicine, oral administration of 25 coral capsules and Danzhen headache capsules; the former 4 capsules once daily and the latter 2 capsules three times daily	To be treated with conventional Western medicine, take flunarizine or thagrelate orally, the former takes one capsule every night, the latter three times a day, the dose is 100 mg	1 month	In the treatment group of 38 patients, 10 were cured, 19 were significantly improved, seven were effective, and two were ineffective. The total effective rate of treatment was 94.7%; in the control group of 38 patients, six were cured, 15 were significantly improved, 10 were effective, and seven were ineffective. The total effective rate of treatment was 81.6%	Liu et al. (2016a)
Vertigo	Ershiwuwei Shanhu pills	160 cases: 88 males and 72 females	Randomized controlled trials	100 cases: 56 males and 44 females	60 cases: 32 males and 28 females	Boiled blister suit, 1 g once, 1 day	*Gastrodia elata* Blume capsules, 4 capsules at 1 time, 3 times at 1 day	1 month	The total effective rate of the treatment group was 85.00%. After ridit analysis of the comparison results between the two groups, the therapeutic effect of the treatment group was significantly better than that of the control group	Liu et al. (2004)
Acute cerebral infarction	Ershiwuwei Shanhu pills	60 cases: 32 males and 28 males	Randomized controlled trials	30 cases: 19 males and 11 females	30 cases: 13 males and 17 females	On the basis of the control group, add 1 g of Ershiwuwei Shanhu pills once a day	Aspirin 100 mg, once daily; clopidogrel hydrogen sulfate tablets 75 mg, once daily; atorvastatin calcium tablets 40 mg, once daily; ligustrazine 120 mg, once daily; cytidine sodium 0.5 g, once daily; and edaravone 30 mg, twice daily	—	In the control group, there were nine cases with a decrease in NIHSS score, four cases with an increase in NIHSS score, and 17 cases with no change in NIHSS score; in the treatment group, 21 cases showed a decrease in NIHSS score, two cases showed an increase in NIHSS score, and seven cases remained unchanged in the NIHSS score; compared with the control group, the NIHSS score significantly decreased after treatment	Zhu et al. (2020)
Acute cerebral infarction	Ershiwuwei Shanhu pills combined with aspirin	80 cases: 43 males and 37 females	Randomized controlled trials	40 cases: 22 males and 18 females	40 cases: 21 males and 19 females	On the basis of the control group, Ershiwuwei Shanhu pills were given 1 g/time, 1 time/day	Take atorvastatin tablets orally before bedtime, 20 mg/dose, once a day; oral aspirin enteric-coated tablets 100 mg/time, once a day.	60 days	Both groups showed an increase in MMSE scores, a decrease in NIHSS scores, FIB, D-dimer, and platelet aggregation index, with significant changes observed in the observation group	Tan (2020)
Cerebral infarction	Ershiwuwei Shanhu pills	60 cases: 38 males and 22 females	Randomized controlled trials	30 cases	30 cases	Ershiwuwei Shanhu pills, 2 capsules each time, 2 times a day, taken orally, or once a day, 4 capsules each time, taken orally	Huoxue Tongmai tablets, 4 tablets each time, 3 times a day, taken orally.	20 days	Among the 30 cases in the treatment group, six were basically cured, 13 were significantly improved, 10 were improved, and one was ineffective, with a total effective rate of 96.7%; among the 30 cases in the control group, one case was basically cured, eight cases were significantly improved, 12 cases were improved, and nine cases were ineffective. The total effective rate was 70.0%	Wang (2003)
Cerebral infarction	Ershiwuwei Shanhu pills	90 cases: 52 males and 38 females	Randomized controlled trials	45 cases: 27 males and 18 females	45 cases: 25 males and 20 females	Cerebral infarction Tibetan medicine Ershiwuwei Shanhu pills, 2 capsules each time, 2 times a day, taken orally	Huoxue Tongmai tablets, 2 capsules each time, 2 times a day, taken orally.	20 days	Observation group: Among 45 cases, 30 were significantly effective, 13 were effective, and two were ineffective, with a total effective rate of 95.56%; Control group: Among 45 cases, 13 were significantly effective, 19 were effective, and 13 were ineffective, with a total effective rate of 71.11%; after treatment, the NIHSS scores of both groups decreased, and the NIHSS scores of the observation group were significantly lower than those of the control group	Zeng (2019)
Cerebral hemorrhage	Shanhu Qishiwei pills	4 cases	Randomized controlled trials	—	—	Seventy flavored pills of coral, 1 pill per day	—	20 days	The patient's symptoms have decreased. CT scan shows circular low-density lesions visible in the intracranial region. After being discharged from the hospital, the patient took 70 flavored pills of coral under guidance, and their symptoms have improved significantly thus far without any other adverse reactions	Bian (2012)
Refractory heart failure	Heart failure mixture combined with Shanhu Qishiwei pills	150 cases: 90 males and 60 females	Randomized controlled trials	100 cases	50 cases	Conventional anti-heart failure treatment should, in principle, discontinue the use of Western medicine to dilate the coronary artery and improve myocardial ischemia. In severe cases, basic Western medicine treatment such as cardiotonic, diuretic, and vasodilation should be given. At the same time, one pair of heart failure mixture was given daily, after boiling twice, take 500 ml of the medicinal solution and take it warm in two separate doses. Take Coral 70, once a day in the morning, one pill each time, and take it with warm water	Routine anti-heart failure treatment	1 month	Among the 100 cases in the treatment group, 56 were significantly effective, 32 were effective, and 12 were ineffective, with a total effective rate of 88%; among the 50 cases in the control group, 20 were significantly effective, 18 were effective, and 12 were ineffective, with a total effective rate of 76%	He et al. (2007)
Hypertension	Ershiwuwei Shanhu pills	30 cases: 16 males and 14 females	Randomized controlled trials	30 cases: 16 males and 14 females	—	Soak in water in the morning and take it every night while sleeping, once a day	—	1 month	26 cases were cured, accounting for 86.7%; three cases showed significant effect, accounting for 10.0%; one case was ineffective, accounting for 3.3%; total effective rate was 96.7%	Li (2010a)
Hypertension	Ershiwuwei Shanhu pills in combination with dipine drugs	90 cases	Randomized controlled trials	45 cases	45 cases	Combined Tibetan medicine Ershiwuwei Shanhu pills, taken orally with warm water on an empty stomach, 1 capsule/time, 1 dose/day, 30 days as a course of treatment	Treatment with dipines	3 days	The effective rate of the treatment group (95.56%, 43/45) is higher than that of the control group (77.78%, 35/45), and the systolic and diastolic blood pressure after treatment in both groups are lower than before treatment, and the reduction in the treatment group is more significant	LI and Liu (2021)
Cough with lung heat	Ershiwuwei Shanhu pills	54 cases: 33 males and 21 females	Randomized controlled trials	—	—	Coral Ershiwuwei Shanhu pills 1/2, 3 times before meals, powder, 3 times after meals	—	15 days	15 days of recovery	Yang (2003)
Waist, hand, and foot injuries	Ershiwuwei Shanhu pills	17 cases: 11 males and 6 females	Randomized controlled trials	—	—	Take Coral Ershiwuwei Shanhu pills orally, 3 times a day, 1 pill each time, chew carefully and take with boiling water. Take the medicine half an hour before meals. For external use, use 6 pills of Ershiwuwei Shanhu pills at the end, soak in 3 liang of Baijiu, half an hour later, apply externally to the affected part, 3–4 times a day, with moderate force	—	7 days	All 17 cases recovered	Yang (2003)

#### 6.2.1 Clinical application of preparations that contain coral for nervous system disease

Neurological disorders consist of two main areas. First, it is manifested in the treatment of epilepsy disorders. Epilepsy is a chronic disease of sudden, transient, recurrent central nervous system malfunction caused by abnormal over discharge of neurons in the brain (Xu et al., 2009). Ershiwuwei Shanhu pills can cause a significant reduction in the number of seizures, shorten the duration of seizures, improve the type of seizures, reduce the symptoms of headache after seizures, and reduce the degree of cognitive impairment, with significant anti-seizure and anticonvulsant effects. Clinically, 112 patients were randomly divided into a treatment group and a control group, and the treatment group was given 25 coral pills, whereas the control group was treated with Western standardized AEDs. The results showed that the total effective rate of the treatment group was 91.07%, whereas that of the control group was only 67.86% (Wang et al., 2014a). In the treatment of patients with epileptic tonic–clonic seizures, the total effective rate of the treatment group (taking Ershiwuwei Shanhu pills alone) was 88.23% (Wang et al., 2013b). The effects of combination drug treatment regimens have also been reported. Patients were treated orally with Ershiwuwei Shanhu pills in combination with oral levetiracetam tablets or carbamazepine, and the results showed that the therapeutic effect was higher than that of conventional Western medical treatment, reducing the levels of serum IL-2, TNF-α, sICAM-1, IL-6, and CRP. The combination of drugs has better clinical efficacy in the treatment of epilepsy, while improving the immune function of patients and reducing the inflammatory response (Huang and Zhao, 2017; Yuan et al., 2018).

Migraine, tension headache, and intractable headache are common clinical primary headache disorders. Sixty-three patients with migraine were randomly divided and treated with either Ershiwuwei Shanhu capsules or Nao Zhen Ning. After 30 days, 30 out of 33 patients taking Ershiwuwei Shanhu capsules were effectively treated, with a total effective rate of 90.9%, and 22 out of 30 patients taking Nao Zhen Ning were effectively treated, with a total effective rate of only 73.3% (Renwang and Renqing, 2010). A total of 110 patients were selected for the study, and the efficiency of the treatment group (taking Ershiwuwei Shanhu pills alone) was 94.55%, which was significantly higher than the total efficiency of the control group (taking flunarizine hydrochloride capsules combined with amitriptyline hydrochloride tablets), which was 74.55%. Meanwhile, clinical efficacy observation shows that Ershiwuwei Shanhu pills can improve the clinical outcomes of headache by reducing the abnormal blood flow condition (Wang et al., 2013c). In addition to medication, acupuncture can also be combined with treatment. A total of 110 patients were randomly divided into two groups: the control group was treated with acupuncture, and the observation group was treated with Ershiwuwei Shanhu pills. The results showed that the total effective rate was 80% in the acupuncture group but 94.5% in the observation group. Further study found that β-EP, NO, and 5-HT levels in the observation group were higher than those in the acupuncture group, and ET levels in the observation group were lower than those in the acupuncture group, suggesting that Ershiwuwei Shanhu pills can improve neuro-endocrine factors and regulate cerebral blood flow rate in patients with migraine, thereby contributing to the improvement of migraine symptoms (Gu, 2014). As early as 2000, a study found that Ershiwuwei Shanhu pills combined with acupuncture could treat intractable headaches (Bai and You, 2000). Modern research has shown that Ershiwuwei Shanhu pills not only dilate blood vessels and improve the effect of microcirculation in the brain but also alleviate the symptoms of vascular smooth muscle spasm to restore local central cerebral area blood perfusion, thereby relieving headache symptoms (Li, 2007; Yang, 2010).

#### 6.2.2 Clinical application of preparations that contain coral for cardiovascular and cerebrovascular diseases

In cardiovascular system diseases, it is effective in treating poststroke headache and cerebral infarction-related conditions. Sixty-four patients with poststroke headache were studied, and after 4 weeks of treatment, the efficiency of the treatment group who underwent conventional medical treatment in combination with Ershiwuwei Shanhu capsules was 93.75%, which was significantly higher than that of the control group who underwent only conventional medical treatment (56.25%). The patient’s headache level is reduced; the number of attacks is significantly reduced, and the duration of headache is significantly shortened during the treatment period (Shi and Zheng, 2018). In a study by Dongmei Guan, the clinical efficacy of Ershiwuwei Shanhu capsules given to patients with poststroke headache was higher than that of the reference group. Pharmacological analysis showed that the mechanism was similar to that of primary headache, which acted by dilating blood vessels, regulating cerebral blood flow, and improving neurological function (Wang and Li, 2014; Yang et al., 2015).

Regarding cerebral infarction disease, 90 patients were randomly divided into the control group and the observation group and were given Huoxue Tongmai Pian and Ershiwuwei Shanhu pills, respectively. The results showed that the efficacy of Erxuoyi Coral Pill was better, and its clinical application was more valuable (Zeng, 2019). On the basis of the study that Ershiwuwei Shanhu pills can significantly reduce infarct foci in rats with focal cerebral ischemia, researchers randomly selected 60 patients and tested their blood lipid, uric acid, homocysteine, and other levels. The results showed that the treatment group had elevated levels of glutamate transaminase, glutamic oxaloacetic transaminase, and other enzymes, which clearly demonstrated the efficacy of Ershiwuwei Shanhu pills in treating cerebral infarction, but such pills have a certain effect on heart, liver, and kidney function, and the mechanism may be related to the regulation of blood lipids (Zhu et al., 2020). Although aspirin can improve the hypercoagulable state of blood, the drug alone is not effective. Patients with acute cerebral infarction were observed after using Ershiwuwei Shanhu pills combined with aspirin, and the control group used aspirin combined with atorvastatin. The results showed that MMSE scores increased; NIHSS scores, FIB, D-dimer, and platelet aggregation index decreased, and the changes were large in the observation group (Tao et al., 2022). Pharmacological studies have further shown that Ershiwuwei Shanhu pills can inhibit cerebral thrombosis, reduce the area of cerebral infarction, reduce brain tissue edema, dilate cerebral blood vessels, and improve cerebral blood circulation and brain tissue metabolism, which coexist with the antithrombotic effect of aspirin to improve the therapeutic effect and have higher clinical application value (Tan, 2020). The total effective rate of Shanhu Qishiwei pills for the treatment of persistent heart failure also reached 88%, whereas no significant toxic side effects were found (He et al., 2007).

Ershiwuwei Shanhu pills cured 26 out of 30 cases of hypertension, with a total efficiency of 96.7%. The pharmacological study proved that the whole formula lowered blood viscosity, reduced water retention in the body, and changed blood rheology. It has a long-lasting and stable effect on lowering blood pressure level, which is more effective for unstable hypertension (Li J. G., 2010). In addition, combination treatment regimens not only improve treatment efficiency but also ensure treatment safety. The total effective rate of Ershiwuwei Shanhu pills combined with diphenhydramine drugs in the treatment of hypertensive patients was as high as 95.56%, which was higher than that of patients taking only diphenhydramine drugs, whose effective rate was only 77.78% (LI and Liu, 2021).

#### 6.2.3 Clinical application of preparations that contain coral for orthopedic system diseases

Similar to the application of coral single medicine, compound prescription is also effective in orthopedic system diseases, and it has better efficacy in the clinical treatment of neurogenic cervical spondylosis, lumbar myofasciitis, and traumatic synovitis of the knee joint (Li W. H. et al., 2013; Jiao et al., 2013). In 65 clinical cases of neurogenic cervical spondylosis, after taking Ershiwuwei Shanhu pills orally combined with acupuncture based on the condition for one course of treatment, the pain symptoms were significantly reduced, and after two courses, the symptoms disappeared completely, and no recurrence was seen thus far (Zhang and Zhang, 2011). Ershiwuwei Shanhu pills have also been used in combination with conventional Western medical treatment. The researchers randomly assigned 84 patients to the control group who received flunarizine hydrochloride capsules orally and the observation group received Ershiwuwei Shanhu capsules in combination with flunarizine hydrochloride capsules. The results showed that the observation group could increase patients’ plasma neurohypophyseal hormone concentration, reduce pain, and improve blood flow velocity in the vertebral and basilar arteries, with a final total effective rate of 90.48%, which is significantly higher than the 69.05% of the control group (Ren et al., 2015). A patient with lumbar myofasciitis was treated with oral and external application of Ershiwuwei Shanhu pills for 20 days; all the symptoms were removed, and no recurrence was observed after 1 year of follow-up (Li, 2006).

#### 6.2.4 Clinical applications of preparations that contain coral for other diseases

In addition, 25 flavored coral pills have shown clinical return in trauma, herpes zoster, and respiratory system. Seventeen patients with lumbar, hand, and foot sprains and smash injuries were cured within 7 days by using coral Ershiwuwei Shanhu pills alone, internally and externally on the affected area (Yang, 2003). In clinical practice, the efficacy of taking Ershiwuwei Shanhu pills as the monarch drug, together with Chouluo Gengsheng powder, in clearing heat and detoxifying, clearing, and moistening the lung in 54 cases of patients with lung fever obtained satisfactory results (Yang, 2003). Acyclovir is also used clinically in combination with Ershiwuwei Shanhu pills to treat herpes zoster, a neuropathic pain caused by damage after the activation of the herpes zoster virus, which belongs to the Tibetan medical term “albichoriasis” (Zhang H. Y., 2012). Therefore, the treatment of neuralgia of herpes zoster with Ershiwuwei Shanhu pills has unique effects and efficacy. Shanhu Qishiwei pills is also a common classical compound prescription containing coral and is used to treat cerebral hemorrhage, limb paralysis, epilepsy, and various neuritis. In four patients with cerebral hemorrhage, headache and vomiting were relieved after taking Shanhu Qishiwei pills once a day for 20 days, and round-like hypodense foci were observed in the skull. In addition, no other adverse effects were observed (Bian, 2012). Although the compound prescriptions are diverse and the ingredients that exert their medicinal effects may be multiple, the synergistic effect of the coral in treating the symptoms of the disease and improving the efficacy of the treatment is evident.

In clinical practice, we use one side to treat multiple diseases, identify the syndrome accurately, and use the right medicine for the syndrome. The conventional Western medical treatment package includes symptomatic treatment, such as improving the patient’s hemodynamics and pain relief, but the efficacy is not significant (Ren et al., 2015). The therapeutic rate of combined drugs is higher than that of single or compound drugs, and it can even produce additional therapeutic effects. Therefore, it has a higher promotion value and is an effective solution worth promoting in the clinic.

## 7 Discussion

Coral is an important marine biological resource, and species resources are extremely confusing and complex. In the Qing dynasty (1,616–1912 AD), red coral is a symbol of official status. In India and Tibet of China, people use coral as an auspicious object to worship Buddha, mostly to make Buddhist beads and decorate the statue of the deity in the temple (Hong, 2009). In ancient records, coral applications in medicine have also long been recorded, but only a few have pointed out that coral in medicine is a combination of coral species for the use of red coral. However, red coral has a broad range and many species, such as *Corallium japonicum* Kishinouye^※^, *Corallium secundum* Dana^※^, and *Corallium elatius* Ridley. *Corallium japonicum* Kishinouye^※^ (trade name: Aka) is mostly used in compounding. Arca is expensive, but no reports have been retrieved on whether other red corals can be substituted. In addition, the corals studied in modern pharmaceutical research involve a total of 34 families and 99 genera of corals, dominated by the families Alcyoniidae, Nephtheidae, and Plexauridae^※^. Coral species are confusing and complex, and sorting out their resource species not only helps us distinguish corals but also lays the foundation for developing new drugs and further research on corals.

Coral has a long history of medicinal value, which can remove corneal opacity, improve eyesight, tranquilize the mind, promote wound healing, and stop bleeding. Modern pharmacological studies have also gradually verified the medicinal value of coral and its mechanism of action. First, coral transplantation in the human body does not cause rejection; in coral, countless fine pores will gradually grow microscopic blood vessels and synthesize living cells of the bone. Numerous studies have reported that coral has become an alternative material to bone, and coral is often used in the fields of maxillofacial surgery and orthopedics (Guillemin et al., 1989; Zhu, 2001; Lai, 2017). Second, active ingredients such as terpenoids (diterpenes and sesquiterpenes) and steroids extracted from coral have evident pharmacological properties such as antiviral, antibacterial, antioxidant, and antimalarial activities. In addition, some of the active ingredients show not only good enzyme inhibition activity but also evident anticonvulsant, antiepileptic, and sedative–hypnotic effects in the nervous system; in the cardiovascular system, they show anti-tubular formation activity and proangiogenic activity as well as a certain amount–effect relationship. The antihypertensive, hypolipidemic (Chun et al., 2022), and antiulcer (Elshamy et al., 2017) activities have also been relevantly verified. Most of these chemical compounds were extracted from corals of Alcyoniidae and Gorgonidae^※^, and the compounds extracted from a particular coral may have multiple uses. Therefore, the study of active ingredients in corals has become the cornerstone of subsequent pharmacological studies, and exploring the mechanism of action of active substances can be a research direction to provide a basis for the elucidation of pharmacological effects and the design of clinical experiments (Wang, 2015).

Coral has various pharmacological activities, among which cytotoxic, anti-inflammatory, and analgesic pharmacological effects are more prominent. A549, HL-60, MCF-7, colon cancer cells, K562, HeLa, and other tumor cells are research hotspots. Scholars have mostly evaluated the inhibitory and apoptotic effects of different concentrations of active ingredients on different cells by MTT assay and SRB method. Studies have also shown that cytotoxicity can be influenced by compound structure. For example, prostaglandins with hydroxyl groups have good inhibitory properties (Hurtado et al., 2020); sterols introduced with hydroxyl groups decrease the inhibitory potency against HeLa cell lines; and acetyl groups increase the cytotoxic activity. Pro-inflammatory enzymes, particularly iNOS for nitric oxide production and prostaglandin-producing COX-2, play a central role in inflammatory mechanisms (Wei et al., 2013). In addition, glial cells and elastin are also important components of the anti-inflammatory mechanism. At present, pharmacological experiments of coral have identified its active ingredients. However, most of the results of pharmacological studies are derived from cellular or animal models, and they do not fully prove their effectiveness, so more clinical trials are needed to confirm these findings (Zhang X. L. et al., 2015).

Clinically, coral is often processed into powder for punching or used directly to treat bone diseases, in addition to showing good therapeutic effects in the treatment of epilepsy, primary headache, migraine, cerebral infarction, hypertension, neurogenic cervical spondylosis, and lumbar myofasciitis. In the face of complex diseases, obtaining the desired effect of a single drug is difficult, so coral is often used in combination with other drugs to treat the disease, which has satisfactory results in clinical applications. At a certain efficacy, compound prescriptions containing coral exhibit the same effects as when coral used alone. However, given the large number of herbs contained in the compound, the role played by coral remains unclear; the effect may be weakened; the effect may be synergistically enhanced; or another effect may be stimulated. Moreover, the mechanism of action of coral remains unknown; thus, further research is needed.

Although coral toxicity is not included in the *Pharmacopoeia of the People’s Republic of China*, studies have found that coral toxicity is mostly found in marine ornamental soft corals of the Zoanthidae family. Palytoxin is the main toxic compound. Nonpeptide neurotoxins were extracted from water coral all of which have toxic effects on the skin, cornea, etc. Short-term minor injuries are reversible with medication or emergency measures, with only a few disabilities or a significant decrease in quality of life because of sequelae. No significant acute toxicity was observed in coral-related compound preparations, but if applied for a long time, toxicity to the liver, kidneys, lungs, and other internal organs can still occur in a dose-dependent manner. The toxicity of coral is not yet generalized because of the complexity and diversity of its species. Coral insects are toxic, but whether coral is toxic after calcification is yet to be studied because of the special nature of coral.

The organic compounds in corals are remarkably studied, whereas other compounds, such as trace elements, are less studied. Coral as mineral medicine should strengthen the exploration and development of other compounds, such as trace elements, to pave the way for improving its quality standards and research on the basis of medicinal substances. Toxicological studies have also come to the forefront. The limited clinical trials are not perfect in quality, but they still have some reference value, and more scientific and representative clinical trials are needed in the future. At present, coral is used in several different fields, such as medical and apparel (Zhang Q. Y., 2013), with more areas still under development. Its value in medical care is particularly significant, which needs more attention and extensive research.

Coral reefs are one of the most diverse ecosystems on Earth, sensitive and fragile marine ecosystems, and one of the most sensitive environmental indicators of global climate change (Huang et al., 2023). Coral reefs not only provide a place for many fish and marine invertebrates to lay eggs, reproduce, and avoid predators, but also have extremely high biodiversity. It also plays a crucial role in homeland security. At present, habitat loss, diseases, bleach accidents, and species invasion are the main causes of coral death (Ma et al., 2018). *Antipatharia*, *Scleractinia*, *Helioporacea*, *Gorgonaceae*, *Tubiporidae*, *Corallium*, *Corallium elatius, Corallium japonicum*, *Corallium konjou*, and *Corallium secundum* are officially listed as endangered. Fishing for any coral species may have some impact on the ecosystem, as coral reefs are a complex ecosystem that is interdependent on many other organisms. So wild acquisition is strictly prohibited for species that have already been listed as extinct in the wild, regionally extinct, and critically endangered. Strengthening the protection and management of rare and endangered wild plants of vulnerable, near-endangered, and non-endangered species by building dynamic monitoring databases and strictly implementing on-site conservation measures is essential to maintain ecological balance and biodiversity (Wang, 2023). The most fundamental thing is that the coral species (extinct in the wild, regionally extinct, critically endangered, and rare and endangered wild plants of vulnerable, near-endangered, and non-endangered species) included in the list have no distinction between beneficial and insignificant and should receive equal protection. Strict implementation of the Endangered Species Law should help them restore to their normal numbers.

It is worth noting that some corals are currently listed as endangered on CITES and IUCN lists, such as *Antipatharia* ^※^, *Tubiporidae* ^※^, *Corallium elatius* ^※^, *Corallium japonicum* ^※^, *Corallium konjou* ^※^, and *Corallium secundum* ^※^, ensuring that a balance between the sustainability of scientific research and the protection of endangered species is a complex but crucial task. The development and research on the effectiveness of coral is likely to lead to the indiscriminate capture of coral, thereby exacerbating the endangered situation of coral. Ethically speaking, the application and development of coral should be prohibited. This article mainly discusses the endangerment caused by coral medicinal use. Therefore, we encourage scientists to use new technologies and methods, such as remote sensors, remote sensing technology, and genetic analysis, to reduce interference with endangered species while providing more valuable data (Wang et al.). The most effective measure is to avoid and prohibit the use of coral. We call on scholars to devise strategies for replacing coral in treatments to alleviate and solve the problems of endangerment of corals. First, search for alternatives based on similar biological species relationships. Species that are closely related often have similar physiological structures, as well as their chemical composition and pharmacological activities (Tian et al., 2023). *Naemorhedus goral* and *Saiga tatarica* have similar chemical components such as proteins, peptides, and amino acids (Liu et al., 2018), and as a substitute, *Naemorhedus goral* has a better sedative effect (Jiang and Zhai, 2006). Therefore, soft corals, sea fans, and sea yellows with similar chemical components have become one of the ways to replace endangered corals. Second, search for alternatives based on similar pharmacological effects. On the one hand, coral is usually used as an orthopedic material for the treatment of bone diseases. Therefore, we can achieve the same therapeutic effect by using other composite materials such as composite resin, ceramic, rubber, organic glass resin, and metal alloy instead of coral. This measure can not only achieve the effect of treating diseases but also reduce the use of coral. On the other hand, based on the bioactive substances extracted from coral, search for other organisms contain similar bioactive substances. For example, the pharmacological activity of cetosane diterpenoids can be extracted from *Boswellia carterii* (Xu et al., 2023). Medicinal compounds such as terpenoids and other substances can be extracted from *Croton tiglium* and *Panax notoginseng* (Wei et al., 2022). In modern research, replacing bile powder of *Rhinoceros unicornis* with that of *Bubalus bubalis* and using other animal bile powder instead of that of *Selenarctos thibetanus* as medicine have alleviated the endangerment problem of animals to a certain extent (Bai et al., 2018; Chen et al., 2022; Ye et al., 2022). Third, search for alternatives based on artificial domestication and breeding. Artificial cultivation of coral has become a feasible method (Wang, 2020). The artificial breeding technology of coral can be divided into sexual reproduction and asexual reproduction and can be classified into *in situ* cultivation technology and off-site cultivation technology according to the cultivation environment. The South China Sea Institute of Oceanography, Chinese Academy of Sciences, has carried out experiments on coral sexual reproduction and coral larva cultivation and proliferation in Sanya Bay and Yongxing Island in Xisha, Hainan Province. At present, it has mastered the reproductive law and larva development process of species such as *Acropora gemmifera* ^※^ and *Platygyra sinensi*s (Yu et al., 2022)^※^. Fourth, look for alternatives based on synthetic methods. The active ingredients with pharmacological activities can be directly synthesized by chemical synthesis and enzyme engineering technology. The development of artificial *Moschus berezovskii* can be described as a sword sharpened for decades, which has completely solved the problem of long-term shortage of *Moschus berezovskii* supply (Fu et al., 2023). Artificial *Panthera tigris* is similar to natural *Panthera tigris* in fingerprint, pharmacological and pharmacodynamic indexes, and clinical efficacy (Liu and Han, 2006). They are all used in a variety of Chinese patent medicines (Yan et al., 2023). Finally, find the alternative mode of biotechnology based on industrialization. The use of coral stem cells to cultivate medicinal parts and secrete metabolites is a biotechnology method that can meet the requirements of syngeneic and homogeneous substitutes. Animal stem cell research mainly focuses on the direct use of stem cells for disease treatment, repair of organ damage, and establishment of the drug screening platform (Shi, 2020). *Taxus* *chinensis*’s stem cells have been used to produce paclitaxel and its corresponding active substance (Lee et al., 2010), and *Panax ginseng* stem cells have been used to produce ginseng (Jang et al., 2023).

Although some developments have not been broken yet through due to incomplete research on the material foundation and mechanism of action, as well as immature artificial farming techniques, which have prevented the formation of large-scale farming, modern biotechnology and multi-omics detection methods have brought new avenues for the development and evaluation of animal drugs, using genomics, proteomics, transcriptomics, metabolomics, and other detection methods at the molecular and cellular levels. The balanced and comprehensive approaches such as implementing multidimensional and multi-level systematic evaluation at the animal level, establishing new approaches for alternative research, and providing support for the protection, development, and utilization of endangered medicinal animals help ensure the survival of endangered species while providing valuable knowledge for the scientific community (Chun et al.).

It is undeniable that effective scientific research can alleviate the problem of endangerment of species, but the decision to “protect” and/or “establish a recovery plan” does not depend only on science. At the same time, there is ambiguity among managers regarding governance issues, and the institutional management plan seems to have failed to address the vulnerability of endangered species. Therefore, a statutory plan should be established to fundamentally alert people. Unfortunately, only relatively few countries have enacted national legislation on endangered and threatened species. Internationally, the Endangered Species Act of 1973 is a legislative model. Its implementation has achieved significant results, and it is said that 90% of the species on the bill’s protected list have been restored (Teng and Zhang, 2022). So the government and relevant departments should strengthen regulations and policies to ensure that research on endangered coral species does not lead to abuse or overfishing, such as restrict or prohibit fishing and destructive activities and adopt sustainable fishing practices, including limiting fishing quantities, using selective fishing nets, and monitoring fishing activities, to protect marine ecosystems. Obviously, effective protection of endangered and threatened species in the ocean depends on appropriate legislation developed to protect them, and similarly, achieving the goals of these legislative tools depends on the political and social factors that affect their implementation. Since the 21st century, the debate between the protection of endangered species and economic benefits and ecological values has never stopped. Congress and the government always try to weaken the effectiveness of bills and tend to choose economic development. Opponents believe that some measures have harmed their own interests, and even more so, some are testing the “red line” for potential benefits. However, the public is more inclined to support the protection of endangered species, and the public support rate for the bill remains high. Obviously, the importance of protecting species is higher than economic growth and protecting private property, and maintaining biodiversity is a global cause (Jeffrey, 2016). Wildlife managers have a greater responsibility to ensure that their management actions reflect public values and attitudes. Third, the negative impact of social media comments on the public is very strong, which leads to significant differences in public beliefs in participating in endangered species management (Rodgers and Willcox, 2018). Some people, even with an urgent desire to protect endangered species, have low mobility. Therefore, it is important to raise awareness among the public, governments, and businesses about the importance of protecting coral reefs, encourage environmental action, and alleviate the pressure faced by these fragile ecosystems. In addition, with the development of industrialization and technology, the marine ecosystem is increasingly deteriorating, the ability to accommodate and rescue wildlife is weak, and habitats are gradually lost. Scientific research and monitoring are necessary. This includes ecosystem monitoring, which regularly monitors the health status of coral reef ecosystems and changes in environmental parameters such as temperature, salinity, and acidity, as well as research on coral diseases. Studying the pathogenesis of coral diseases will help develop prevention and control strategies (Yang, 2021). It is also necessary to reduce the flow of land pollution into the ocean, including agricultural and urban sewage discharge, as well as pollutants from rivers and streams (Gao, 2023; Zhu and Hu, 2023).

There is relatively little research on linking wildlife value orientations with attitudes toward T&E species and non-charismatic species, and even to a large extent, it has been overlooked (George et al., 2016). We encourage cooperation among scholars, conservation organizations, and governments to work together to achieve the common goals of scientific research and conservation (Lv et al., 2020; Zou and Jiang, 2023).

Coral is one of the marine species. The imminent extinction of coral reminds people to take immediate measures to protect endangered species and the ecological environment. The substitution principles are as follows: search for alternatives based on similar biological species relationships. Search for alternatives based on similar pharmacological effects. Search for alternatives based on artificial domestication and breeding. Look for alternatives based on synthetic methods. Search for the alternative mode of biotechnology based on industrialization. These principles should be applied to protect all organisms from natural sources and not restricted to corals. Among them, the alternative strategy of artificial domestication is one of the most fundamental and effective measures. For example, treatments using pearls, centipedes, and others should be supplemented and replaced by other resources to prevent their endangerment or even extinction. In addition, reducing pollution, which means taking measures to reduce marine pollution, especially plastic waste, agricultural and industrial emissions, oil pollution, and sustainable economic management methods including fisheries, tourism, and marine technology, can promote economic growth and create decent employment opportunities, thereby reducing people’s hunting of certain species, which is also one of the effective measures to protect ecosystems (Chinese and Foreign Experts and Scholars Talk Together on Marine Ecological Protection in the Process of Modernization, 2023). Protecting endangered species and protecting ecosystems is our mission and responsibility. These measures can balance the goal of protecting ecosystems and meeting human needs.

Recently, the signing of the High Seas Treaty in September 2023 and the Nagoya Protocol in October 2010 has pushed the protection of endangered species and ecological environmental issues onto the international stage, marking a significant shift in ecological governance from disorder to order. The Chinese government must strictly abide by the agreement, and in addition, it can educate the Chinese people, enterprises, and other relevant departments through media and policies to reduce and prohibit the use of coral. China’s participation should make good use of the platform for treaty and agreement consultations and be more proactive in clarifying the content and value of this theory to the international community, injecting new vitality into the protection of ecology and subsequent consultations. The feasibility of global action is low, and its effectiveness is difficult to assess and regulate. However, the ability of countries or regional international organizations to respond more quickly to environmental changes greatly contributes to the improvement of the marine environment. International treaties can not only regulate the behavior of contracting parties but also promote the practice of non-contracting parties. Conducting this work under international cooperation undoubtedly has the most legitimacy and credibility. This not only strengthens cooperation among countries and encourages them to jointly address high seas protection issues but also enhances people’s awareness of coral protection and ecosystem protection through universal participation. At the same time, these agreements also limit people’s indiscriminate killing of corals and causing damage to the environment, pointing the way for protecting the ecological environment. To a large extent, it is urgent to alert and call on people to protect corals and endangered species, but how to achieve from regional practice to universal participation is still a difficult implementation dilemma.

Warning: The coral species marked with "※" have been included in the rare species list of CITES and IUCN. We should respect life and nature. We should protect wild animals and create a safe home for them. In addition, we call on researchers to increase their attention and importance to the protection of endangered corals and to conduct limited and valuable coral-related research within the framework of domestic and foreign laws and regulations. We also call on non-researchers to refrain from illegally collecting and using endangered corals after reading this review article and to carry out collection and utilization activities within the framework of domestic and foreign laws and regulations.

## 8 Conclusion

Marine invertebrates, a rich potential source of drug precursors, have been a popular avenue for the international search for drugs or drug precursors in recent decades. In the last two decades, coral chemistry and pharmacology research has made some achievements and discovered some new compounds with unique structures and strong physiological activities, but the utilization of corals is limited to only a small number and species of families, such as Alcyoniidae, Nephtheidae, Plexauridae, and Gorgoniidae^※^ *.* This article provides the first comprehensive account of six aspects of the medicinal history, species, chemical composition, pharmacological activity, toxicology, and clinical application of coral in China. Coral is a natural mineral medicine, and its active ingredients are mixed and difficult to extract and identify. At present, the effective compounds extracted from coral are terpenoids, steroids, and nitrogen-containing compounds, with sesquiterpenes and diterpenes being the main compounds of terpenoids. However, during extraction, extraction conditions, and the joint use of related techniques, such as ICP-MS and LC-MS, have not been reported. Exploring the best extraction of the active ingredients of coral is a breakthrough for future experiments. The pharmacological effects of most of the compounds isolated from coral have been developed. The pharmacological activities of terpenoids are relatively rich, including cytotoxicity, anti-inflammatory, antibacterial, and antiviral. Second, steroid compounds also play important roles in antitumor, anticancer, and anti-inflammatory activities. Finally, other compounds such as lipids and aromatic compounds play important roles in their pharmacological activities such as antioxidant and immunosuppressive effects. However, they mostly reside in superficial areas, which shows a long way to go in the study of the mechanism. In addition, scientists are encouraged to use new technologies and methods, such as remote sensors and gene analysis, to reduce interference with endangered species while providing more valuable data. Toxicological studies have shown that corals of the family Zoanthidae cause toxic reactions in people through contact and inhalation, but they can be treated with pharmacological relief. With regard to clinical application, coral is mostly used in combination with other drugs to treat diseases, with limited cases of coral alone, which may lead to the inability to prove the effectiveness of coral but is still informative. Pharmacological studies of coral are mostly about the monomer extracted from coral, whereas clinical studies are more about the compound prescription application of coral. Studies show that coral is often used as a substitute for orthopedic materials to treat diseases such as bone defects and bone hyperplasia. Compound preparations that contain coral are widely used in the treatment of neurological diseases such as migraine, primary headache, epilepsy, cerebral infarction, hypertension, and other cardiovascular and cerebrovascular diseases.

More extensive and in-depth research on the active ingredients of coral and its mechanism of action should be focused on deepening the understanding at genetic and molecular levels in the future to make it better applied in practice. In addition, whether the absorption, distribution, metabolism, and excretion as well as the blood concentration of coral change over time after administration in the body remains unclear. Coral is often used in the form of powder into the body, but whether coral powder can be taken orally as well as differences and similarities between its oral and external therapeutic effects remains unknown. Finally, diluted coral extracts have been derived as new drugs for the treatment of diseases. Therefore, the form of coral intake should not be limited to powder or as an orthopedic material. However, the development of its active ingredients is not a research strategy and prospect. It provides different ideas for the development of new drugs. We experienced pressure and challenge during the study of the clinical application of coral but offered us a good opportunity (Ai et al., 2006).
